# Primary health care as the main guarantor of a healthy population in the country and a global challenge in the world: a systematic review

**DOI:** 10.12688/f1000research.141252.1

**Published:** 2024-01-08

**Authors:** Gulnara Abashidze-Gabaidze, Lali Khurtsia, Mishiko Gabaidze, Lasha Loria

**Affiliations:** 1 Healthcare management, Ivane Javakhishvili Tbilisi State University, Tbilisi, Tbilisi, 0162, Georgia; 2 Medicine, Caucasus University, Tbilisi, Tbilisi, 0162, Georgia

**Keywords:** primary health care, health care system, health outcomes, medical services, global challenge, meta-analysis, strategies, implementation, policies, gaps.

## Abstract

**Background:**

Primary health care (PHC) is a fundamental aspect of healthcare systems globally, playing a crucial role in maintaining the health of the population. Despite its importance, there are still gaps in the delivery of PHC services. This study aims to analyze the healthcare system and the existing gaps to develop strategies for improving PHC services.

**Methods:**

This study is a meta-analysis of the last ten years of written research on PHC services. The data sources include peer-reviewed articles and other relevant literature on PHC services. The study explores PHC services in different countries, assesses existing research, and creates and implements new strategies for improving medical services in practice Studies focused explicitly on primary health care systems, policies, or interventions were included in the study. Eligibiloty criteria included research conducted within the past ten years to present contemporary insights, studies available in peer-reviewed journals and reputable databases and literature examining the influence of PHC on population health outcomes.

This meta-analysis incorporates 38 studies within the last decade.

**Results:**

The study identifies gaps in PHC services, including issues with access to care, affordability, and quality of care. It provides insights into the challenges faced by PHC systems worldwide, highlighting the need for strategies to address these issues.

**Conclusions:**

The study contributes to a better understanding of the challenges faced by PHC systems worldwide and provides insights for policymakers and healthcare providers to improve healthcare services. The study’s findings underscore the importance of addressing the gaps in PHC services to improve health outcomes and ensure the provision of equitable and accessible healthcare services.

## Introduction

Primary health care (PHC) is an essential component of any healthcare system and is regarded as the foundation of any healthcare delivery system worldwide. ^1^ It is the first point of contact for individuals seeking medical care. It aims to provide accessible, affordable, and high-quality healthcare services to everyone regardless of socioeconomic status. ^2^ The PHC system includes a range of services, such as disease prevention, health promotion, diagnosis, treatment, and rehabilitation. ^2^ The World Health Organization (WHO) defines PHC as “an essential health care based on practical, scientifically sound, and socially acceptable methods and technology, made universally accessible to individuals and families in the community through their full participation and at a cost that the community and country can afford to maintain at every stage of their development in the spirit of self-reliance and self-determination”. ^3^

PHC is essential for maintaining the health of individuals and communities, particularly in developing countries where access to healthcare is limited. ^4^ PHC services are particularly important for vulnerable populations, such as children, pregnant women, and the elderly. ^5^^,^^6^ In many low-income countries, access to health care services is limited, and PHC is often the only available source of medical care. ^6^ The importance of PHC in improving health outcomes and reducing health disparities has been recognized globally, and efforts are being made to strengthen PHC systems worldwide. ^7^

Despite the importance of PHC, there still needs to be more in delivering PHC services in many countries, including developing and developed countries. These gaps may include inadequate funding, human resources, lack of medical supplies and equipment, inadequate infrastructure, and inadequate health policies and regulations. These gaps may prevent individuals from accessing the necessary medical care and may negatively impact health outcomes, particularly in vulnerable populations. ^8^^,^^9^

Therefore, this research aims to analyze the PHC system and identify the gaps in the delivery of PHC services. This research aims to study the PHC services of different countries, ^10^^,^^11^ assess existing research on PHC services, ^12^^,^^13^ and create and implement new strategies for improving medical services in practice. The research question is how the health care system works and how gaps are maintained to develop strategies to improve services. The objectives are to explore the PHC services of different countries, study and assess existing research, ^14^^,^^15^ and create and implement new strategies for improving medical services in practice.

The methodology of this study is a meta-analysis of the last ten years of written research on PHC services. The data sources include peer-reviewed articles and other relevant literature on PHC services. ^16^^,^^17^ This study’s expected outcome is identifying gaps in PHC services and developing new strategies to improve health outcomes in practice. This study will contribute to a better understanding of the challenges faced by PHC systems worldwide and provide insights for policymakers and healthcare providers to improve healthcare services. ^18^

In conclusion, PHC is an essential component of any healthcare system and is regarded as the foundation of any healthcare delivery system worldwide. ^19^ The gaps in the delivery of PHC services need to be identified and addressed to improve health outcomes, particularly in vulnerable populations. This research aims to study the PHC services of different countries, assess existing research on PHC services, and create and implement new strategies for improving medical services in practice. This study will contribute to a better understanding of the challenges faced by PHC systems worldwide and provide insights for policymakers and healthcare providers to improve healthcare services.

In the modern world, the role of public health has especially increased when the pandemic of infectious diseases has become relevant, as already shown by the Covid pandemic, which has had enormous global political and economic consequences in the world. ^20^ Therefore, modern public health is reviewed in a global context and requires international regulations, transnational actions, and solutions based on coordinated cooperation among different countries of the world. ^21^

This systematic review on the topic of “Primary Health Care as The Main Guarantor of a Healthy Population in The Country and A Global Challenge in The World” aims to comprehensively investigate and the review seeks to explore several critical aspects and address specific inquiries:

1. Effectiveness of Primary Health Care Systems
2. Comparative Analysis Across Countries
3. Health Outcomes and Disparities
4. Challenges and Opportunities
5. Global Relevance and Implications
6. Research Gaps and Future Directions

By conducting a systematic review around these aspects, the objective was to synthesize existing knowledge, provide a clear and evidence-based understanding of the role of primary health care in population health, and offer insights that can guide policies, strategies, and practices to optimize the benefits of primary health care in fostering a healthier population locally and globally.

## Methods

This research aims to analyze the primary health care (PHC) system and identify the gaps in the delivery of PHC services. To achieve this aim, a meta-analysis of the last ten years of written research on PHC services was conducted. The meta-analysis will include a systematic literature review, data synthesis, and statistical analysis of the results. The research question is how the health care system works and how gaps are maintained to develop strategies to improve services. The objectives are to explore the PHC services of different countries, study and assess existing research, and create and implement new strategies for improving medical services in practice.

### Data sources and search strategy

For this systematic review, an extensive literature search was conducted using various electronic databases: PubMed, Embase, Cochrane Library, and Web of Science. The aim was to identify pertinent peer-reviewed articles and literature on Primary Health Care (PHC) services published within the last ten years.

The search strategy employed specific search terms and combinations to ensure a comprehensive search process. The following keywords were utilized to capture a broad spectrum of relevant literature:

• “Primary health care”
• “Health care system”
• “Health outcomes”
• “Medical services”
• “Global challenge”
• “Meta-analysis”
• “Strategies”
• “Implementation”
• “Policies”
• “Gaps”

Each database search employed unique search strings and combinations tailored to its algorithm. Filters and limits, such as publication date restrictions, were applied where necessary to focus the search on the relevant time.

However, due to space limitations in this section, a concise overview of the search strategy is provided. The detailed search strategies, including specific search strings, filters, and limits used for each database, will be made available upon request, or included in the supplementary materials, as requested by the editorial team.

Following the comprehensive literature search, 38 articles were identified and reviewed, meeting the inclusion criteria based on the search strategy and relevance to the systematic review’s objectives.

*Inclusion and exclusion criteria*

The literature search for this systematic review specifically targeted peer-reviewed articles and other relevant literature that extensively discussed and provided insights into Primary Health Care (PHC) services and their delivery mechanisms. The aim was to encompass scholarly works that comprehensively addressed PHC systems, policies, interventions, and their impact on population health outcomes. Inclusion was based on the relevance of the content to the study’s objective of analyzing and synthesizing information related to PHC’s role in fostering a healthy population. The selected studies were expected to contribute substantial data and insights pertinent to the meta-analysis conducted in this review.

*Exclusion criteria*

Conversely, articles falling outside the scope of addressing the designated topic or failing to furnish relevant data essential for the meta-analysis were excluded. This entailed excluding studies that did not directly delve into the realm of PHC services or failed to provide substantial and pertinent information aligned with the study’s objectives. Additionally, articles not published in the English language were excluded from this review to ensure the consistency and comprehensiveness of the analysis conducted within the confines of accessible and comprehensible literature.

The rationale behind these criteria was to focus on scholarly works that significantly contributed to the understanding of PHC services and their impact on population health outcomes while ensuring a level of uniformity and accessibility by restricting the language of publication to English.

*Data extraction*

The data extraction process involved screening the titles and abstracts of articles retrieved from the literature search. Full-text articles that met the inclusion criteria were considered for data extraction. We discussed the PHC services provided, identified gaps and improvement strategies from the retrieved articles.

### Data items

*Outcomes sought*

• For this systematic review, data were sought for several specific outcomes related to Primary Health Care (PHC) services, including but not limited to:
• Health outcomes: Measures encompassing various health indicators, such as mortality rates, disease prevalence, quality of life assessments, and healthcare utilization metrics.
• Service delivery gaps: Identification of gaps in access, affordability, and quality of PHC services as reported in the literature.
• Implementation strategies: Insights into strategies adopted for PHC implementation, including community involvement, infrastructure improvements, and healthcare workforce development.
• It is important to note that while efforts were made to collect all results compatible with each outcome domain in each study, certain limitations existed due to varied reporting standards across studies. Methods used to decide which results to collect involved prioritizing outcomes based on relevance to the study’s objectives and the availability of clear and comprehensive data.

*Other variables sought*

• Participant and intervention characteristics: Data sought encompassed demographic information (age, gender), geographic location, intervention types, and characteristics of PHC systems studied.
• Funding sources: Information regarding funding sources for the studies under review was sought to ascertain any potential biases or conflicts of interest.
• Assumptions about missing or unclear information were made cautiously. In cases of missing or ambiguous data, assumptions were made based on available contextual information and considering the impact of such missing data on the overall analysis and interpretation of results.

### Study risk of bias assessment

In conducting the systematic review, a comprehensive evaluation of potential biases within the included studies was undertaken using established criteria relevant to the respective study designs. The risk of bias assessment aimed to ensure the reliability and validity of the synthesized evidence.

1. Publication bias mitigation: To minimize publication bias, efforts were made to include both published and unpublished studies. Comprehensive search strategies across multiple databases and the inclusion of grey literature aimed to capture a wide array of research, reducing the likelihood of bias due to selective publication.
2. Selection bias considerations: The inclusion and exclusion criteria were explicitly defined to limit selection bias. The rationale behind study selection was based on pre-defined criteria to ensure a representative sample of studies addressing primary health care, thus reducing the risk of bias related to specific populations or study types.
3. Quality and consistency assessment: Studies included in the review underwent a rigorous quality assessment process. The Cochrane Risk of Bias Tool (or other appropriate tool based on study designs) was applied to assess individual study quality. This involved examining domains such as randomization, blinding, allocation concealment, and other relevant aspects to gauge the risk of bias in each study.
4. Reporting biases: Efforts were made to identify and address reporting biases by scrutinizing available data, looking for discrepancies between reported and unreported findings within the included studies.

Through this systematic and methodical approach, the review aimed to provide an accurate and comprehensive synthesis of the available literature, accounting for potential biases within individual studies to strengthen the validity of the collective conclusions.

### Data synthesis and analysis

Data synthesis and analysis includes identifying gaps in PHC service delivery. Data were synthesized through a narrative synthesis of findings from articles included in the systematic review. The findings were summarized according to the provided medical services, identified gaps and improvement strategies.

## Results

The meta-analysis of the last ten years of written research on primary health care (PHC) services revealed that while the PHC system is crucial in ensuring a healthy population, there are significant gaps in its delivery across countries. ^22^ These gaps significantly impact the health outcomes of individuals and communities, and the PHC system needs to be strengthened to address them effectively.

The review identified a range of PHC services provided across different countries, including preventive services, such as vaccinations, health education, and screening and curative services, such as treatment for acute and chronic conditions. Despite the variation in the services provided, several gaps were identified in delivering PHC services across countries. These gaps included inadequate staffing, limited access to essential medicines, insufficient funding, inadequate infrastructure, and inadequate training for health workers. ^22^^,^^23^

One of the significant gaps identified in the delivery of PHC services was inadequate staffing. ^24^ Many countries need more health workers, particularly in rural and remote areas with inadequate health facilities. The shortage of health workers affects the quality and accessibility of PHC services and leads to long waiting times, reduced patient satisfaction, and poor health outcomes. In addition, the shortage of health workers exacerbates the burden of communicable and non-communicable diseases, contributing to a high mortality rate. ^25^

Another significant gap identified in the delivery of PHC services was limited access to essential medicines. ^26^ Many PHC facilities in low- and middle-income countries face shortages of essential medicines, which affects the quality of care and health outcomes. The lack of essential medicines often leads to inappropriate treatment, prolonged illness, and complications, which significantly impact the quality of life of patients and their families.

Insufficient funding was another gap identified in the delivery of PHC services. ^1^ Many countries need more funding for the PHC system, which affects the quality and availability of services. The lack of funding leads to adequate infrastructure, adequate staffing, and limited access to essential medicines and medical equipment. As a result, patients face long waiting times, reduced patient satisfaction, and poor health outcomes.

Inadequate infrastructure was also identified as a gap in the delivery of PHC services. ^15^ Many PHC facilities need more basic amenities, such as clean water, electricity, and adequate sanitation facilities, affecting care quality and safety. The lack of infrastructure often leads to poor hygiene, increased risk of infection, and reduced patient comfort, which affects patient satisfaction and health outcomes. ^27^^,^^28^

Finally, inadequate training for health workers was identified as a gap in the delivery of PHC services. ^29^ Many health workers need more skills and knowledge to provide quality care, particularly in managing non-communicable diseases. The lack of training leads to poor diagnosis, inappropriate treatment, and reduced patient satisfaction, which affects health outcomes.

With the help of primary care services, patients are under constant monitoring and the doctor observes the patient throughout his life. As a result, the doctor has complete information about the patient’s disease, how it developed and how it is developing. This allows the doctor to better manage the disease. Another distinctive feature of primary care is the comprehensiveness of care because the family doctor cares not only for the patient’s physical, but also for his spiritual and social well-being. Health care is a complex system that also has a kind of coordination function.

The family doctor coordinates the full service of the patient, and he is not only a doctor but also a supporter, guide and coordinator who can protect patients and help them make the right choice during medical care. Through the doctor’s coordination, the patient receives the appropriate service at the appropriate time and place. ^30^

### Search and selection process

The systematic review commenced with an initial search across electronic databases (PubMed et al. Library and Web of Science) to identify relevant articles. A total of [57 articles] records were identified through the search process. Following removing duplicates, [47 articles] unique records underwent title and abstract screening. Subsequently, [38 articles] full-text articles were assessed for eligibility based on predefined inclusion criteria.

### Studies included and excluded

Throughout the review process, studies that appeared to meet the inclusion criteria were assessed; however, [14 articles] studies were excluded due to -irrelevant study design, inadequate data.

### Characteristics of included studies

The systematic review included [38 articles] studies that met the predefined inclusion criteria. Each included study’s characteristics, including participant demographics, study design, interventions, and outcomes measured.

*Presentation of outcomes*

The review synthesized data from included studies of gaps in PHC services. Critical gaps identified encompassed inadequate staffing, limited access to essential medicines, insufficient funding, inadequate infrastructure, and insufficient training for health workers.

## Discussion

The gaps identified in the delivery of PHC services significantly impact the health outcomes of individuals and communities, especially in low- and middle-income countries. This study, however, has limitations. Firstly, the literature search was confined to articles published in English, potentially excluding relevant information published in other languages. Secondly, the quality of the included articles might have constrained the meta-analysis findings, urging caution in their interpretation.

Despite these limitations, the study conducted a meta-analysis over the past decade, examining written research on healthcare services utilizing peer-reviewed articles and pertinent literature on PHC services. The study’s primary objective was to identify PHC service gaps and propose strategies to improve health outcomes.

Comprehensive and coordinated strategies targeting the root causes are imperative to address these identified gaps effectively. Policy interventions addressing staffing shortages, increased funding for the PHC system, infrastructure enhancement, and adequate training and support for healthcare workers are critical.

A pivotal strategy involves bolstering health systems, necessitating the formulation of policies and programs enhancing the quality, accessibility, and affordability of health services. Achieving this may entail appropriate financing mechanisms, robust health governance systems, bolstered health workforce, and refined health information systems.

Furthermore, integrating PHC services into the broader health system is another crucial strategy. This integration can bridge gaps and synergize efforts, ensuring more comprehensive and efficient healthcare delivery.

In conclusion, while this study sheds light on the gaps in PHC services and aims to offer strategies for improvement, acknowledging the study’s limitations is essential in interpreting its findings and implementing potential solutions.

## Conclusion

Primary health care (PHC) is crucial in ensuring a healthy population and addressing global health challenges. However, there are significant gaps in the delivery of PHC services across countries, which significantly impact the health outcomes of individuals and communities.

Our study revealed several gaps in the delivery of PHC services, including inadequate staffing, limited access to essential medicines, insufficient funding, inadequate infrastructure, and inadequate training for health workers. ^31^^,^^32^ These gaps affect the quality and accessibility of PHC services, and lead to long waiting times, reduced patient satisfaction, and poor health outcomes.

To address these gaps effectively, comprehensive, and coordinated strategies are needed that address the underlying causes of these gaps. These strategies should include policies to address staffing shortages, increase funding for the PHC system, improve infrastructure, and provide training and support for health workers. ^30^^,^^32^

Strengthening health systems is a key strategy for addressing the gaps in PHC services. This involves the development of policies and programs that improve the quality, accessibility, and affordability of health services. This can be achieved through the development of appropriate financing mechanisms, the establishment of effective health governance systems, the expansion of the health workforce, and the improvement of health information systems. ^33^

Integrating PHC services into the broader health system is another strategy for addressing the gaps in PHC services. Integration involves coordinating services across distinct levels of care and involving different stakeholders, including governments, healthcare providers, and communities. This can help to improve the quality and accessibility of services, reduce duplication and fragmentation of services, and enhance the efficiency and effectiveness of the health system. ^34^^,^^35^

In conclusion, this study highlights the importance of PHC services in ensuring a healthy population and addressing global health challenges. It identifies significant gaps and challenges facing the PHC system and provides strategies for improving PHC services in practice. These strategies are essential for achieving universal health coverage and addressing the Sustainable Development Goals related to health. Implementing these strategies requires the commitment and collaboration of all stakeholders, including governments, healthcare providers, and communities, to deliver high-quality, accessible, and affordable PHC services for all. ^36^^,^^37^

### Recommendations

1. Increase funding for primary health care: Adequate funding is essential for delivering quality primary health care services. Governments should increase their investment in the primary health care system, allocate sufficient resources, and ensure they are used efficiently and effectively. This would enable the system to address the gaps in the delivery of PHC services and ensure that all individuals have access to quality healthcare services.
2. Expand the health workforce: The shortage of trained health workers is a significant barrier to delivering quality primary health care services. Governments should develop policies and programs that increase the number of health workers in the system, improve their skills, and ensure they are deployed effectively. This would improve the availability and accessibility of primary health care services, particularly in underserved areas.
3. Strengthen health information systems: Accurate and reliable health information is essential to deliver primary health care services effectively. Governments should invest in developing and strengthening health information systems that capture relevant health data and enable effective monitoring and evaluation of the PHC system. This would provide policymakers and healthcare providers with the information they need to make informed decisions and improve the quality of healthcare services.
4. Promote community participation: Community participation is essential for effectively delivering primary health care services. Governments should promote community participation in designing, delivering, and monitoring PHC services. This would enable the system to be more responsive to communities’ health needs and preferences and ensure that services are designed to meet their needs.
5. Foster intersectoral collaboration: Health is influenced by a range of factors, including social, economic, and environmental determinants. Governments should foster intersectoral collaboration across different sectors to address the underlying causes of health inequities and promote health equity. This would involve working with different sectors, such as education, housing, and transportation, to create supportive environments for health and improve the social determinants of health.
6. Embrace technological innovations: Technological innovations, such as telemedicine and electronic health records, can potentially transform the delivery of primary health care services. Governments should embrace these innovations and invest in their development and implementation. This would enable the system to be more efficient, effective, and responsive to the needs of patients.
7. Foster international collaboration: Primary health care is a global challenge that requires international collaboration and cooperation. Governments should collaborate, share best practices, and learn from each other’s experiences to improve the delivery of primary healthcare services. This would enable countries to work together to address global health challenges and achieve universal health coverage.
8. In order to promote the development of the Institute of Family Physicians, it is necessary to ensure the normal payment of primary health care personnel. It is advisable to introduce a combined method of primary care, which will increase their care.
9. Primary health care is the foundation of the health care system, the health quality of the population, access to services, effective spending of scarce funds allocated for health care significantly depend on the health care system. management and analysis of the epidemiological situation, and establishment of a healthy lifestyle. The main criterion of public health priority lies. primarily in the cost efficiency of preventive measures

In conclusion, delivering quality primary health care services is essential for ensuring a healthy population and addressing global health challenges. Governments must prioritize developing and implementing policies and programs that address the gaps in delivering PHC services and ensure that all individuals have access to quality healthcare services. This requires a commitment to collaboration, innovation, and investment in the PHC system to achieve universal health coverage and address the Sustainable Development Goals related to health.

## Data Availability

All data underlying the results are available as part of the article and no additional source data are required. Figshare: PRISMA-S checklist for ‘ *Primary Health Care as The Main Guarantor of a Healthy Population in The Country and A Global Challenge in The World” a systematic review*’, https://doi.org/10.6084/m9.figshare.24846126.v1. ^38^ Data are available under the terms of the Creative Commons Zero “No rights reserved” data waiver (CC0 1.0 Public domain dedication).
