# Primary health care as the main guarantor of a healthy population in the country and a global challenge in the world: a systematic review

**DOI:** 10.12688/f1000research.141252.2

**Published:** 2024-10-10

**Authors:** Gulnara Abashidze-Gabaidze, Lali Khurtsia, Mishiko Gabaidze, Lasha Loria

**Affiliations:** 1 Healthcare management, Ivane Javakhishvili Tbilisi State University, Tbilisi, Tbilisi, 0162, Georgia; 2 Medicine, Caucasus University, Tbilisi, Tbilisi, 0162, Georgia

**Keywords:** primary health care, health care system, health outcomes, medical services, global challenge, meta-analysis, strategies, implementation, policies, gaps.

## Abstract

**Background:**

Primary health care (PHC) is a fundamental aspect of healthcare systems globally, playing a crucial role in maintaining the health of the population. Despite its importance, there are still gaps in the delivery of PHC services. This study aims to analyze the healthcare system and the existing gaps to develop strategies for improving PHC services.

**Methods:**

This study is a mixed method a combination of documentary analysis and narrative synthesis as an alternative to meta-analysis. For our systematic review, we primarily focused on articles published within the last 10 years. However, we also included some older articles (published between 2003 and 2009) that provided valuable insights into the historical context and evolution of primary healthcare systems. Data of each article reviewed during the review - Study, Article, Study setting, Study design, Study assessment, Study suggestions are presented in Table 1. A total of 38 articles were reviewed. The data sources include peer-reviewed articles and other relevant literature on PHC services. With appropriate keywords.

**Results:**

The study identifies gaps in PHC services, including issues with access to care, affordability, and quality of care. It provides insights into the challenges faced by PHC systems worldwide, highlighting the need for strategies to address these issues.

**Conclusions:**

The study contributes to a better understanding of the challenges faced by PHC systems worldwide and provides insights for policymakers and healthcare providers to improve healthcare services. The systematic review, which focuses on PHC, was conducted following the PRISMA guidelines. The PRISMA diagram of study selection was used to illustrate the process of article inclusion and exclusion. Table 1 provides a summary of the key information from the selected articles.

## Introduction

Despite rapid advancements in medical technology worldwide, including in Georgia, alarming medical statistics reveal a persistent gap between technological progress and overall population health. Approximately 80% of global deaths are attributed to preventable chronic diseases, underscoring the critical need for effective prevention strategies. Primary healthcare (PHC) is widely regarded as a cornerstone in addressing this issue. This systematic review aims to comprehensively examine existing research on the role of PHC in improving population health, identify common challenges, and propose recommendations based on international best practices. By understanding the factors influencing PHC effectiveness, we seek to contribute to enhanced population health outcomes.

The World Health Organization recommends evaluating health systems using a primary healthcare approach. Evidence suggests that 90% of key universal health coverage (UHC) interventions could be implemented through a PHC framework, potentially saving 60 million lives and increasing average global life expectancy by 3.7 years by 2030. By sharing the findings, knowledge, and best practices from this systematic review, we can collectively assess the effectiveness of various PHC models and work towards a shared vision for improving population health (WHO-PHC-2023).

As a developing country, Georgia has undergone numerous reforms in its PHC system. However, PHC remains underdeveloped. To address these challenges and promote a healthier population, it is crucial to learn from the experiences of other countries and collaborate with global health organizations to develop the most effective recommendations.

Twenty-five years have passed since healthcare reforms began in Georgia, yet an effective primary healthcare (PHC) system has not been fully established. In 2000, the Department of Primary Health Care was created within the Ministry of Health. A year later, it merged with the Department of Public Health to better integrate public health initiatives. However, the success of these efforts has been limited. Low patient referrals to family doctors and a general lack of trust in primary care providers remain significant challenges. ^1^

One key indicator of the underdevelopment of Georgia’s PHC system is the low number of referrals to outpatient medical institutions, with only 2.3 visits per capita, compared to up to 7.5 in European countries. This disparity highlights the insufficient development of primary care in Georgia. As a result, self-medication is widespread, driven by a lack of confidence in primary care services. This situation has led to a concerning outcome: medicinal costs now account for a catastrophically high 40% of total healthcare expenditures in Georgia, compared to 10-15% in European countries.

Given these challenges, it is crucial to analyze the strategies employed by other countries through our systematic review and develop joint recommendations to improve PHC in Georgia.

Therefore, our systematic review aimed to examine and evaluate the experiences of different countries with primary health care (PHC) models, focusing on [specific countries or regions] between [start date] and [end date]. By analyzing these experiences, we sought to identify valuable recommendations for improving PHC systems. Consequently, our research questions were:

Is a well-structured PHC a guarantor of a healthy population?

What are the relationships between PHC and health outcomes in various countries? Our review sought to conduct a detailed analysis based on existing studies to inform policy and practice.

Primary health care (PHC) is an essential component of any healthcare system and is regarded as the foundation of any healthcare delivery system worldwide. ^2^ It is the first point of contact for individuals seeking medical care. It aims to provide accessible, affordable, and high-quality healthcare services to everyone regardless of socioeconomic status. ^3^ The PHC system includes a range of services, such as disease prevention, health promotion, diagnosis, treatment, and rehabilitation. ^3^ The World Health Organization (WHO) defines PHC as “an essential health care based on practical, scientifically sound, and socially acceptable methods and technology, made universally accessible to individuals and families in the community through their full participation and at a cost that the community and country can afford to maintain at every stage of their development in the spirit of self-reliance and self-determination”. ^4^

PHC is essential for maintaining the health of individuals and communities, particularly in developing countries where access to healthcare is limited. ^5^ PHC services are particularly important for vulnerable populations, such as children, pregnant women, and the elderly. ^6^^,^^7^ In many low-income countries, access to health care services is limited, and PHC is often the only available source of medical care. ^7^ The importance of PHC in improving health outcomes and reducing health disparities has been recognized globally, and efforts are being made to strengthen PHC systems worldwide. ^8^

Despite the importance of PHC, there still needs to be more in delivering PHC services in many countries, including developing and developed countries. These gaps may include inadequate funding, human resources, lack of medical supplies and equipment, inadequate infrastructure, and inadequate health policies and regulations. These gaps may prevent individuals from accessing the necessary medical care and may negatively impact health outcomes, particularly in vulnerable populations. ^9^^,^^10^

Therefore, this research aims to analyze the PHC system and identify the gaps in the delivery of PHC services. This research aims to study the PHC services of different countries, ^11^^,^^12^ assess existing research on PHC services, ^13^^,^^14^ and create and implement new strategies for improving medical services in practice. The research question is how the health care system works and how gaps are maintained to develop strategies to improve services. One of the great challenges for the development of primary health care is the accuracy of the production of health statistics in primary health care, as more countries embark on health reforms and strategies to reduce poverty, the need for robust statistics to identify implementation difficulties and evaluate outcomes will become more pressing. Because without it, it is impossible to create a patient-oriented service. ^15^ The objectives are to explore the PHC services of different countries, study and assess existing research, ^15^^,^^16^ and create and implement new strategies for improving medical services in practice. The study emphasizes the importance of implementing comprehensive NCD management strategies that address both prevention and treatment. This could include providing early diagnosis, screening, and treatment for NCDs, as well as promoting healthy lifestyle behaviors and providing support for patients with chronic conditions. ^14^ A study of 18 developed OECD countries found that strong primary healthcare systems are crucial for maintaining population health. Specifically, countries with robust primary healthcare systems experienced a significant 26% reduction in overall mortality rates over three decades. Starfield's primary healthcare scale was used to assess these systems, focusing on both structural and practical aspects. ^17^ The scale's 10 components provide a comprehensive evaluation of primary healthcare, highlighting its essential characteristics. The research conclusively demonstrated that a strong primary care system: Improves health outcomes, Reduces mortality rates and enhances overall population health, Enhances preventive care, Helps prevent diseases and promotes healthy lifestyles, Reduces health disparities, Contributes to more equitable health outcomes across different populations, Improves health system function, Strengthens the overall efficiency and effectiveness of the healthcare system. ^17^

The methodology of this study is a systematic review of the last ten years of written research on PHC services. The data sources include peer-reviewed articles and other relevant literature on PHC services. ^18^^,^^19^ This study’s expected outcome is identifying gaps in PHC services and developing new strategies to improve health outcomes in practice. This study will contribute to a better understanding of the challenges faced by PHC systems worldwide and provide insights for policymakers and healthcare providers to improve healthcare services. In the article, the researchers proposed a useful PHC monitoring tool called the Primary Care Monitoring System (PC Monitor), which can make a certain contribution to the good monitoring of PHC. ^20^

In conclusion, PHC is an essential component of any healthcare system and is regarded as the foundation of any healthcare delivery system worldwide. ^21^ The gaps in the delivery of PHC services need to be identified and addressed to improve health outcomes, particularly in vulnerable populations. This research aims to study the PHC services of different countries, assess existing research on PHC services, and create and implement new strategies for improving medical services in practice. This study will contribute to a better understanding of the challenges faced by PHC systems worldwide and provide insights for policymakers and healthcare providers to improve healthcare services.

In the modern world, the role of public health has especially increased when the pandemic of infectious diseases has become relevant, as already shown by the Covid pandemic, which has had enormous global political and economic consequences in the world. ^18^ Therefore, modern public health is reviewed in a global context and requires international regulations, transnational actions, and solutions based on coordinated cooperation among different countries of the world. ^22^

This systematic review on the topic of “Primary Health Care as The Main Guarantor of a Healthy Population in The Country and A Global Challenge in The World” aims to comprehensively investigate and the review seeks to explore several critical aspects and address specific inquiries:

1. Effectiveness of Primary Health Care Systems
2. Comparative Analysis Across Countries
3. Health Outcomes and Disparities
4. Challenges and Opportunities
5. Global Relevance and Implications
6. Research Gaps and Future Directions

By conducting a systematic review around these aspects, the objective was to synthesize existing knowledge, provide a clear and evidence-based understanding of the role of primary health care in population health, and offer insights that can guide policies, strategies, and practices to optimize the benefits of primary health care in fostering a healthier population locally and globally.

## Methods

**Figure 1.**
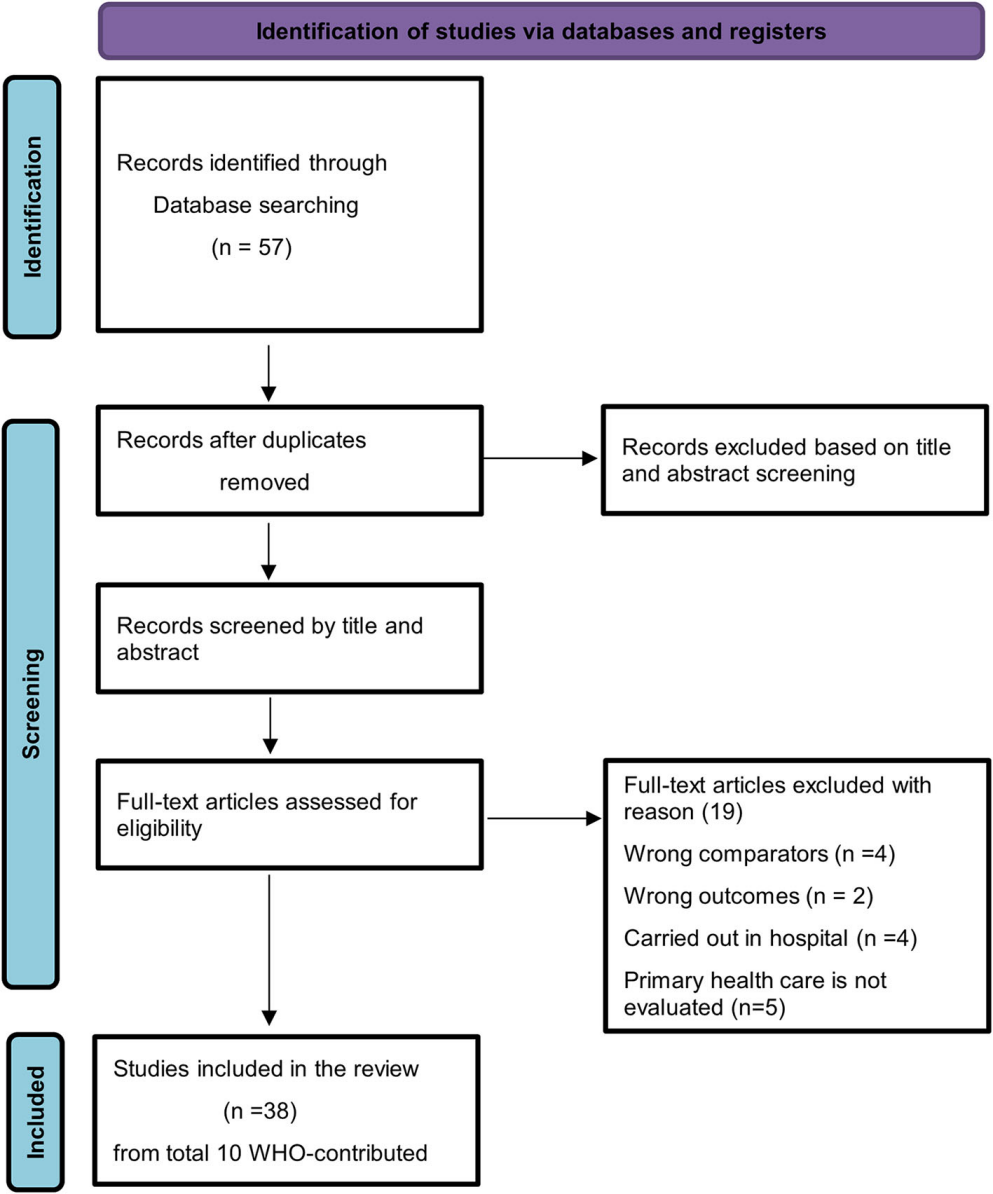
PRISMA diagram of study selection. This work is licensed under CC BY 4.0. To view a copy of this license, visit https://creativecommons.org/licenses/by/4.0/.

**Figure 2.**
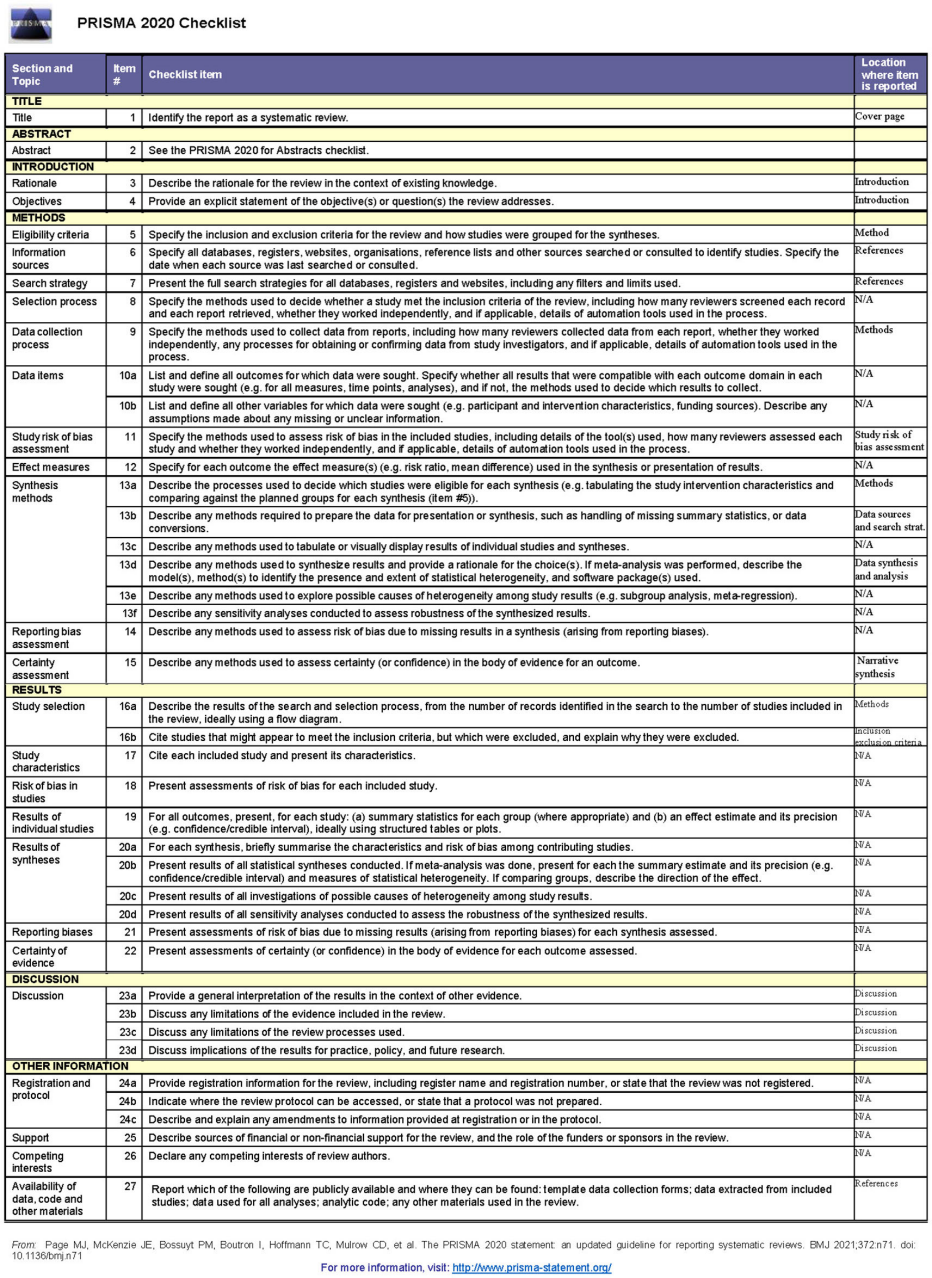
PRISMA 2020 Checklist.

This research aims to analyze the primary health care (PHC) system and identify the gaps in the delivery of PHC services. To achieve this aim, a systematic review of the last ten years of written research on PHC services was conducted. However, we also included some older articles (published between 2003 and 2009) that provided valuable insights into the historical context and evolution of primary healthcare systems (There were only 6 articles in 2003-2009). A systematic review will include a systematic literature review, data synthesis, of the results. We used a mixed method a combination of documentary analysis and narrative synthesis as an alternative to meta-analysis, which has practical value in the development of PHC. The research question is how the health care system works and how gaps are maintained to develop strategies to improve services. The objectives are to explore the PHC services of different countries, study and assess existing research, and create and implement new strategies for improving medical services in practice. Table 1, Figure 1 (Prisma diagram of study selection) and Figure 2 (PRISMA Checklist) are presented for the visibility of the narrative synthesis analysis of the data source considered during the research.

**Table 1.** All discussed articles are included here: Study, Article, Study setting, Study design, Study assessment, Study suggestions to support the reasoning of the systematic review (With the exception of the articles of the World Health Organization).

Study	Article	Study setting	Study design	Study assessment	Study suggestions
(28) Margaret E Kruk, Anna D Gage, Catherine Arsenault, Keely Jordan, Hannah H Leslie at all. DOI: 10.1016/S2214-109X(18)30386-3 2018	High-quality health systems in the Sustainable Development Goals era: time for a revolution	Low-income and middle-income countries (LMICs)	A Systematic Review	This research concludes that care in LMICs is often inadequate, with significant gaps in quality across conditions and countries. Vulnerable populations are particularly affected. They highlight points out systemic deficits in the quality of care, including errors in diagnosis, delayed treatment, and negative patient experiences the challenges of limited availability of laboratory facilities and diagnostic equipment, which hinder patient assessment and diagnosis.	Authors recommend universal health coverage (UHC) as a starting point for improving quality, with a focus on establishing a national quality guarantee and prioritizing the poor
(25) Greg Irving,1 Ana Luisa Neves at all. doi: 10.1136/bmjopen-2017-017902 2017	International variations in primary care physician consultation time: a systematic review of 67 countries	The UK, USA, Australia (a systematic review of published and grey literature in English, Chinese, Japanese, Spanish, Portuguese and Russian languages from 1946 to 2016)	Research included observational studies including cross-sectional studies, surveys and cohorts of consultation length with primary care physicians	The research found that 18 countries representing about 50% of the global population spend 5 min or less with their primary care physicians. The review found associations between consultation length and per capita healthcare spending, primary care physician density, and physician burnout: Which is one of the indicators of poor health outcomes of primary health care services.	The article suggests several recommendations to address the issue of short consultation times in primary care: Increase investment in primary care Improve workforce planning Implement efficient scheduling systems Implement electronic health records Promote team-based care Educate patients
(30) A Cameron MPH at all. https://doi.org/10.1016/S0140-6736(08)61762-6 2009	Medicine prices, availability, and affordability in 36 developing and middle-income countries: a secondary analysis	This study analyzed data from 45 WHO/HAI surveys conducted in 36 countries to assess the availability, affordability, and pricing of medicines in the public and private sectors	This approach combines both quantitative and qualitative research methods	The study assessed - Limited availability: The average availability of generic medicines in the public sector ranged from 29.4% to 54.4% across WHO regions. - Price disparities: Government procurement prices for generic medicines were generally higher than international reference prices, but purchasing efficiency varied significantly. - High patient costs: Private sector patients paid significantly higher prices for both generic and originator products compared to international reference prices. - Unaffordable treatments: Many treatments for acute and chronic illnesses were unaffordable for patients in many countries. - Excessive mark-ups: Wholesale and retail mark-ups for medicines were often excessive, contributing to high prices.	Valuable findings of the study: the study highlights the need for stronger policies and interventions to ensure that medicines are accessible and affordable for all populations. - Promoting generic medicines: Encouraging the use of generic alternatives can help reduce costs. - Implementing alternative financing mechanisms: Exploring options like government subsidies or health insurance can help make medicines more affordable for patients. - Addressing excessive mark-ups: Regulating the pricing of medicines, particularly in the private sector, can help prevent excessive profit margins.
(21) Gregory Sawin at all. https://doi.org/10.1016/j.pop.2019.07.006 2019	Primary Care Transformation	The United States	A Scoping Review	What is the main problem with the current US healthcare system? The US spends the most on healthcare per capita but has poor health outcomes. This could be due to factors like the fee-for-service model that incentivizes more procedures instead of preventive care.	The need for primary care transformation for better primary care: Shared vision: Teams should have a clear focus on patient experience and engagement Empowered staff: Traditional hierarchies may be challenged to enable broader staff interaction with patients. Community-driven needs, Focus on skills-Training, Focus on building skills to support patient empowerment and understanding.
(2) Su et al. BMC Health Services Research https://doi.org/10.1186/s12913-023-09979-3	Evaluating the efficiency of primary health care institutions in China: an improved three-stage data envelopment analysis approach	This study is designed to gauge the efficiency of PHC institutions by using 2012–2020 panel data covering 31 provinces in China. The 2012-2020 timeframe covers a period of significant healthcare reforms and development in China	This study applied an improved three-stage data envelopment analysis (DEA) model to evaluate the efficiency of PHC institutions in China. The traditional DEA models include the CCR model proposed by Charnes, Cooper and Rhodes	The study found: PJD does not have a gatekeeping function, coordination between hospitals and primary health care facilities is broken, referral rate in PJD decreased from 59.7% to 50.2% and increased in inpatients to 45.8%. %. A low level of technology and management was revealed - this is reflected in the deterioration of efficiency indicators: total efficiency (TE) decreased from 0.807 to 0.546, pure technical efficiency (PTE) from 0.870 to 0.594 and efficiency. (SE) from 0.934 to 0.917. This reduction confirms the need for significant improvements to the PJD system.	The study recommends several strategies to strengthen primary healthcare institutions (PHC): Tailored Policies, Environmental Considerations, Scale Efficiency, Human Resource Development, Infrastructure and Technology, Coordination and Collaboration, Continuous Evaluation
(7) Zulfiqar A Bhutta at all. DOI: 10.1016/S0140-6736(08)61407-5 2008	Alma-Ata: Rebirth and Revision 6 Interventions to address maternal, newborn, and child survival: what difference can integrated primary health care strategies make?	They assessed relevant publications from UN agencies, bilateral agencies, non-governmental organisations, research bodies, and academic institutions.they then did two specific case studies from representative countries in Africa (Uganda) and south Asia (Pakistan)	Systematic Reviews	Despite efforts since the Alma-Ata Declaration, progress in primary care for MNCH remains limited, with only 16 countries on track to achieve the fourth MDG. A recent study of interventions covering 68 countries showed a global burden of maternal and child mortality of 97%.	Research has shown that PJD interventions can have a significant impact on MNCH and mortality outcomes. Incorporating evidence-based interventions in these two countries could prevent 20-30% of maternal deaths, 10% of neonatal deaths, and 29-40% of all post-neonatal deaths under 5 years of age. In children of age, strengthening MNCH at the primary health care level should be a priority for countries to achieve their Millennium Development Goals to reduce maternal and child mortality. Five intervention packages (web figure) are identified that can be implemented in first-level facilities through outpatient services. Primary health workers, such as medical and paramedical staff, community health workers and other providers as a result of activation
(32) D Kringos, W Boerma, P Groenewegen, at all. DOI: 10.3399/bjgp13X674422 2013	The strength of primary care in Europe: an international comparative study	27 EU member states, plus Iceland, Norway, Switzerland, and Turkey.	Comparative cross-sectional study	Key Findings: Variations in primary care strength Structural and process dimensions: Primary care strength is influenced by both structural factors (governance, economics, workforce) and process factors (accessibility, comprehensiveness, continuity, coordination) Geographic disparities Financial barriers: Out-of-pocket costs can be a significant obstacle to accessing primary care, especially for vulnerable populations Limited use of technology: Many primary care practices do not fully utilize technology for tasks like prevention, public health activities, and information exchange	Recommendations: Strengthen primary care governance Improve economic conditions Invest in workforce development Enhance accessibility Promote continuity of care Leverage technology: Equip primary care practices with adequate technology to support efficient operations and improve communication Enhance patient-provider relationships
(33) Leiyu Shi, DrPH, MBA, James Macinko at all. DOI: 10.3122/jabfm.16.5.412 2003	The relationship between primary care, income inequality, and mortality in US States, 1980-1995	In US States	cross-sectional design for 4 selected years (1980, 1985, 1990, 1995)	The study found a significant association between income inequality and mortality rates across US states from 1980 to 1995. Additionally, it demonstrated that primary care, particularly family medicine, was negatively associated with mortality rates. These findings suggest that strengthening primary care systems could be an effective strategy to mitigate the negative health effects of income inequality.	Recommendations: - Address income inequality: Implement policies to reduce income inequality and improve social determinants of health. - Invest in primary care: Increase investment in primary care, particularly family medicine, to improve access and quality of care. - To Consider the long-term impact: Recognize the long-term benefits of primary care on population health and plan accordingly. - To Evaluate the role of specialty care: Further research is needed to understand the specific role of specialty care in population health outcomes. - Implement a multi-pronged approach: Address both social determinants of health and healthcare system factors to improve overall population health.
(34) Barbara Starfield DOI: 10.1136/jech.2009.102780 2011	Politics, primary healthcare and health: was Virchow, right?	N/A	N/A	Rudolf Virchow's assertion that medicine is a social science and politics is nothing but medicine on a large scale remains highly relevant in contemporary public health.	A comprehensive approach to healthcare management Continued research and innovation: The article emphasizes the importance of ongoing research and development in healthcare management to develop effective and equitable solutions Focus on primary healthcare
(35) September 23, 2019	“Universal health coverage: moving together to build a healthier world”	N/A	N/A	Limited access to essential health services Catastrophic out-of-pocket health expenditure Inadequate investment: The level of investment in primary healthcare is insufficient to meet the target of universal health coverage by 2030. Health system weaknesses: Many health systems are not sufficiently prepared to respond to the needs of the population, particularly in terms of emerging diseases, non-communicable diseases, and aging populations Limited financial resources Health workforce shortages: There is a global shortage of health workers, particularly in low- and middle-income countries, which limits access to healthcare.	Ways to Improve Primary Healthcare Based on the 2019 UN Declaration: Increased Investment Equity and Inclusion: Ensure that primary healthcare services are accessible to all, regardless of socioeconomic status, gender, or location. Address health inequities and eliminate discrimination in healthcare settings Strengthening Health Systems: Invest in a competent health workforce, improve health infrastructure, and enhance legislative and regulatory frameworks to support primary healthcare Community-Based Approach Disease Prevention and Management Data-Driven Decision Making:Strengthen public health surveillance and data systems to inform evidence-based decision-making and improve healthcare planning.
(23) Berwick DM.at all *Health Aff.* 2002;21(3):80–90. 12026006 10.1377/hlthaff.21.3.80	A user’s manual for the IOM’s “quality chasm” report	United States healthcare system	mixed-methods approach, with some elements of quantitative research	quality of healthcare in the United States: The study assessed the current US healthcare system as fragmented, ineffective and often dangerous. He argues that the system is unable to provide the quality of care that patients expect and deserve due to a variety of factors, including Systemic problems: such as unrealistic reliance on human memory, poor communication systems, and unrealistic demands on human alertness. Organizational factors: such as lack of leadership, inadequate information technology, and ineffective care coordination. Environmental impacts: such as outdated regulations, limited funding, and litigation.	The author offers a number of recommendations for improving the US health care system, which should be applied and considered by other countries as well: Redesigning microsystems: Small units of care must be knowledge-based, patient-centered, and systems-minded. Strengthening Organizations: Healthcare organizations must be better designed to support microsystems and achieve the six goals of improvement. Reform the external environment: The external health environment, including financing, regulation, accreditation, litigation, professional education and social policy, must be more conducive to quality improvement.
(6) Starfield B: New York, NY: **Print ISBN:** 9780195125429 **Publisher:** Oxford University Press	Primary Care: Balancing Health Needs, Services, and Technology	Starfield analyzed data from different health systems around the world to draw conclusions about general principles and best practices in primary care.	mixed-methods approach, with qualitative and observational.	Primary Care: Balancing Health Needs, Services, and Technology" provides compelling evidence for the importance of primary care in achieving optimal health outcomes	By implementing these recommendations, health systems can strengthen primary health care sectors and improve population health and well-being. Increased Investments Strengthened workforce Improved access Integrating Technology Team-based care Community Partnerships Policy support
(9) Shi L at all... Jones & Bartlett Learning, 2017 ISBN 1284175170	Essentials of the U.S. Health Care System	PHC development	mixed-methods approach, with qualitative and observational	Challenges in Primary Care in America: Access to Care - Many rural areas and underserved communities lack sufficient primary care providers, leading to long wait times and limited access Insurance Coverage Cost Heavy Workloads- Primary care providers often face overwhelming patient volumes, limiting their ability to spend adequate time with each patient. Electronic Health Records (EHRs) Value-Based Care Chronic Diseases Work-Life Balance	Recommendations for Improving Primary Healthcare in the United States Support the development of community health centers and rural health clinics Increase funding for medical education and residency programs, especially in primary care specialties. Improve Payment Models Transition to value-based care Enhance Care Coordination Invest in health information technology Promote team-BASED CARE Implement patient-centered medical homes Implement patient-centered medical homes Invest in social programs Integrate social services into primary care Promote health equity Invest in provider education and training
(27) J E Epping-Jordan at all... doi: 10.1136/qshc.2004.010744 2004	Improving the quality of health care for chronic conditions	Developing countries	A Systematic Review	The text discusses the growing burden of chronic conditions worldwide and the challenges faced by healthcare systems in providing adequate care. It highlights the increasing prevalence of non-communicable diseases, mental health disorders, and HIV/AIDS, especially in developing countries. The text also addresses the quality and access issues related to chronic care, particularly for disadvantaged populations.	The text highlights the urgent need for improved healthcare systems to effectively manage the growing burden of chronic disease worldwide. To overcome this challenge, researchers propose a chronic care model (CCM) that benefits from: Self-management support Delivery system design Decision support Improved outcomes: The CCM has been shown to improve the quality of care for patients with chronic illnesses. Wide-scale adoption: Over 1000 healthcare organizations have implemented the CCM. Evidence-based approach: The CCM is based on evidence and has been shown to be effective in various settings.
(37) Khatri *et al. BMC Primary Care* (2023) 24:236 https://doi.org/10.1186/s12875-023-02194-3	People-centred primary health care: a scoping review	A total of fifty-two studies were included in the review; most were from high-income countries (HICs), primarily focusing on patient-centred primary care: 39-(HICs) 19- studies were from the USA 8- studies were from Canada and the Netherlands 6- studies were from the UK and Australia 4- studies were from Norway and Sweden Two studies were from upper-middle-income countries (UMICs), Three studies were from low-and lower-middle-income countries (LMICs)	Systematic Reviews and Meta-Analyses The researchers employed thematic analysis, adopting Gale's framework method.	This scoping review assessed issues and challenges related to people-centred health services in PHC/primary health services. The review showed that patient-centered PHC challenges are lack of doctor visits, lack of discussion, low level of administrative organization, low trust of patients towards health care medical personnel.	To foster patient-centered PHC, several key areas require attention Increased Public Involvement Empathy Development Digital Technology Integration Innovative Funding Models Benefits of Patient-Centered PHC Improved Satisfaction and Well-being Increased Involvement and Adherence
(20) Tengiz VERULAVA at all. Archives of the Balkan Medical Union Copyright © 2022 Balkan Medical Union https://doi.org/10.31688/ABMU.	DEVELOPMENT OF PUBLIC HEALTH IN GEORGIA: CHALLENGES AND POLICY ISSUES	Georgia	Documentary analysis, which included official documents and journal publications.	The text provides valuable historical context by discussing the changes in the post-Soviet healthcare system and highlights the significant role of international organizations in shaping the public health care system of Georgia	The authors offer the following advice: Prioritize public healthThe authors emphasize the importance of placing public health at the forefront of national priorities, particularly in a globalized world where infectious diseases and pandemics pose significant threats Invest in public health infrastructure: The text highlights the need for strong public health systems, including well-equipped laboratories, trained personnel, and effective surveillance mechanisms Strengthen international cooperation Implement evidence-based policies Ensure adequate funding
(10) Tina Janamian at all.. doi: 10.5694/mja14.00295 2014	A systematic review of the challenges to implementation of the full extent of the expanded role of nurses in general practice	Australia	Systematic review of peer-reviewed literature.	The research evaluated the challenges and barriers to implementing the Patient-Centered Medical Home (PCMH) model in Australia Specifically, the article assessed: Challenges with transformation and change management Difficulties with electronic health records (EHRs) Challenges with funding Insufficient practice resources and infrastructure	The research offers several key recommendations Long-term commitment and support Effective change management Investment in resources and infrastructure EHR adoption Care for the education of nurses and their encouragement is necessary because of the shortage of nurses
(11) S. Mendis at all … Volume 2012, Article ID 584041, 7 pages doi: 10.1155/2012/584041	Gaps in Capacity in Primary Care in Low-Resource Settings for Implementation of Essential Noncommunicable Disease Interventions	LMICs (low- and middle-income countries) 2 low income countries: Benin, Eritrea 4 low-middle-income countries: Sudan, Bhutan, Sri Lanka, Vietnam 2 upper middle-income countries: Suriname and Syria	The research method was purposive sampling research method	The study assessed PHC gaps in 4 areas: financing, access to medicines, technological shortages, shortage of medical personnel. Summary of Primary Care Facility Capacities for Non-Communable Diseases (NCDs) Key Findings: Equipment and Supplies-Stethoscopes and sphygmomanometers were widely available (85-90%), but availability of other equipment like ambubags, oxygen masks, nebulizers, ECGs, peak expiratory flowmeters, and pulse oximeters varied significantly Healthcare Workforce-Physicians were present in all PC facilities in only two countries. In others, they were limited or absent. Nurses and health assistants were the primary providers. Medical Records- Paper-based records were the norm, with limited use of electronic systems. Follow-up systems and referrals were often disorganized. Health Financing: Public investment in health is inadequate, eading to high out-of-pocket expenses.	Overall, the study highlights significant gaps in primary care facility capacities for NCD management, particularly in rural areas. Improving access to essential medicines, strengthening the healthcare workforce, and implementing effective medical record systems are crucial for improving NCD care
(12) Vari M Drennan at all.. Southampton (UK): NIHR Journals Library; 2014 May	Investigating the contribution of physician assistants to primary care in England: a mixed-methods study	England	A mixed-methods study conducted at macro, meso and micro organisational levels in two phases: a rapid review, a scoping survey of key national and regional informants, a policy review, and a survey of PAs and comparative case studies in 12 general practices	The impact of including physician assistants in general practice teams on the patients’ experiences and outcomes: High Patient Satisfaction Continuity and Complexity Longer Consultation Times Varying Understanding of PA Role Informational Strategies Patient Outcomes: GPs saw older patients with more comorbidities, while PAs saw more patients with minor symptoms PAs and GPs achieved similar outcomes in terms of reconsultation rates, procedures, investigations, prescriptions, referrals, and over-the-counter medicine advice. More Patient Advice	The study suggests that PAs can be successfully integrated into general practice teams with minimal impact on the organization. However, effective integration requires careful planning, staff education, and ongoing supervision Integrating PAs into PHC will help physicians focus on more complex patients and tasks Future Workforce Needs: The growing demand for primary care services suggests a need for more mid-level staff like PAs. By handling 25% of a general practitioner's workload, physician assistants can contribute significantly to primary care. Their presence alongside nurses can improve the overall quality of services
(13) Rezapour et al. BMC Public Health (2019) 19:911 https://doi.org/10.1186/s12889-019-7237-8	Developing Iranian primary health care quality framework: a national study	Iran	mixed-methods approach combining a literature review - with a qualitative research component.	The study used a rigorous and comprehensive approach to identify and prioritize QIs for evaluating the quality of Iran's PHC system. A Delphi study was conducted with 39 national health professionals to assess and prioritize QIs.	Advice for Other Countries Based on the Iranian Study An Iranian study highlights the importance of developing a Quality Assessment Framework (QAF) for Primary Health Care (PHC) QAFs specifically tailored to each country's unique needs, cultural context and health challenges, which can play a critical role in the continuous assessment and quality improvement of countries' PHC services.
(17) Rifat Atun at all.. 2010;25:104–111 doi: 10.1093/heapol/czp055	Integration of targeted health interventions into health systems: a conceptual framework for analysis	Developing countries	Qualitative and conceptual-research methods	research assessed: Health system characteristics: The governance arrangements, financing mechanisms, planning processes, monitoring and evaluation systems, and demand generation activities within the health system. The context: The broader environment within which the health system operates, including cultural norms, beliefs, and values.	The study provides several recommendations for improving healthcare systems and integrating targeted health interventions: For policymakers Tailor interventions to context Align with global standards Prioritize continuous improvement For healthcare providers: Provide patient-centered care Focus on the needs and preferences of patients, and involve them in decision-making Use technology effectively Invest in human resources Strengthen infrastructure Improve financing Enhance governance: Strengthen the governance of the health system to ensure accountability, transparency, and efficiency.
(19) Kringos et al. BMC Family Practice 2010, 11:81 http://www.biomedcentral.com/1471-2296/11/81	The european primary care monitor: structure, process and outcome indicators	Europe (31 country)	A systematic review	This article discusses the importance of having good data on primary care systems in Europe: Strong primary care systems are linked to better health outcomes, lower costs, and improved coordination of care. The study assessed: governance; economic conditions and workforce development.	In this study, the authors propose a system called the Primary Care Monitoring System (PC Monitor) to collect and compare data on primary care systems across Europe, although it would be useful for all countries. Regular implementation of primary care monitoring (eg, every 5 years) can track trends in primary care over time.
(1) Tengiz VERULAVA at all. https://doi.org/10.31688/ABMU.2022.57.4.07	PRIMARY HEALTH CARE REFORMS IN GEORGIA: THE EXPERIENCE AND CHALENGES	Georgia	Mixed-methods-documentary analysis, which included official docu ments and non-official journal publications.	Analyzing Georgia's Healthcare Reforms and Primary Care Challenges: Underdeveloped Primary Care Self-Treatment High Out-of-Pocket Costs	Recommendations Strengthen Primary Care Infrastructure Promote Preventive Care Implement Financial Protection Mechanisms Improve Patient Education
(14) Kabir A, Karim MN, Billah B. BMJ Open 2021; doi: 10.1136/bmjopen-2021-051961	Primary healthcare system readiness to prevent and manage non- communicable diseases in Bangladesh: a mixed- method study protocol	Bangladesh	Mixed-method design. The study will combine quantitative and qualitative approaches	This study is the first of its kind to comprehensively assess the Bangladesh PHC system for NCD-related services, combining both supply-side and demand-side factors Filling a knowledge gap Comprehensive approach- The study addresses a critical information gap regarding the readiness of the PHC system to address the rising burden of NCDs in Bangladesh. which will be an example for other countries	The authors did not explicitly provide recommendations for improving the management of noncommunicable diseases (NCDs) in this specific study. Strengthen the PHC system: The study highlights the need to enhance the capacity and readiness of the primary healthcare (PHC) system to address NCDs. This could involve investing in infrastructure, human resources, and equipment; improving access to essential services; and strengthening coordination between different levels of care.
(22) Macinko J, Starfield B at all. doi: 10.1111/1475-6773.00149 2003	The Contribution of Primary Care Systems to Health Outcomes within Organization for Economic Cooperation and Development (OECD) Countries, 1970–1998	The OECD, or Organisation for Economic Co-operation and Development (18 Country)	Pooled, cross-sectional, time-series analysis of secondary data using fixed effects regression	This study assessed the contribution of primary health care systems to health diversity in 18 wealthy OECD countries. (OECD) countries for three decades. Key findings: Strong primary care systems are associated with improved population health Countries with stronger primary care systems had lower mortality rates, both overall and from specific causes like asthma, bronchitis, emphysema, pneumonia, cardiovascular disease, and heart disease.	Key conclusions: Primary care is important for population health: Strong primary care systems are associated with improved health outcomes, even after considering other factors. Health reform efforts have focused on primary care: Many OECD countries have implemented health reforms aimed at strengthening primary care. There is room for improvement in primary care: While progress has been made in some areas, many countries still have deficiencies in practice features like coordination and community orientation. Improving primary care can be challenging: Changes to structural features like financing and geographic regulation can be difficult to achieve.
(24) Barbara Starfield DOI: 10.1016/j.gaceta.2011.10.009	Primary care: an increasingly important contributor to effectiveness, equity, and efficiency of health services. SESPAS report 2012	multiple OECD countries ( N90)	The study design was a pooled, cross-sectional, time-series analysis.	Stronger primary care leads to: Reduced hospital utilization 6% fewer inpatient admissions 5% fewer outpatient visits 10% fewer emergency room visits 7% fewer surgeries Lower healthcare costs Better preventive care and early diagnosis	If primary care has a demonstrably salutary impact on health and equity in health, it follows that stronger primary care should produce better outcomes than weaker primary care.
(15) Carla AbouZahr at all. 2007. https://doi.org/10.1016/S0140-6736(07)60463-2	From data to policy: good practices and cautionary tales	N/A	Routinely reported service data	The text discusses the challenges and strategies for effective use of data in policymaking. Evidence-based policymaking. Barriers to evidence-based policymaking Investments needed	Need to ensure effective use of data in policy development: data availability and quality, improve data collection - develop standardized data collection tools and protocols, would be good - invest in data management and analysis, so that data are properly stored, managed and analyzed using appropriate methods, increase capacity to help,Help solve the problem - train health workers and policy makers on data collection, analysis and interpretation.

It is important to note that, since our systematic review focused on primary healthcare (PHC), we considered it relevant to include articles from 2002 to 2008 due to their valuable historical data. Of the 38 articles included, 6 were published between 2002 and 2009, while the remaining 32 were published between 2010 and 2023. Two reviewers participated in the review process to ensure the reliability of inter-evaluator assessments.

### Data sources and search strategy

For this systematic review, an extensive literature search was conducted using various electronic databases: PubMed, Embase, Cochrane Library, and Web of Science. The aim was to identify pertinent peer-reviewed articles and literature on Primary Health Care (PHC) services published within the last ten years.

The search strategy employed specific search terms and combinations to ensure a comprehensive search process. The following keywords were utilized to capture a broad spectrum of relevant literature:

• “Primary health care”
• “Health care system”
• “Health outcomes”
• “Medical services”
• “Global challenge”
• “Meta analysis”
• “Strategies”
• “Implementation”
• “Policies”
• “Gaps”

Each database search employed unique search strings and combinations tailored to its algorithm. Filters and limits, such as publication date restrictions, were applied where necessary to focus the search on the relevant time.

However, due to space limitations in this section, a concise overview of the search strategy is provided. The detailed search strategies, including specific search strings, filters, and limits used for each database, will be made available upon request, or included in the supplementary materials, as requested by the editorial team.

Following the comprehensive literature search, 38 articles were identified and reviewed, meeting the inclusion criteria based on the search strategy and relevance to the systematic review’s objectives.

*Inclusion and exclusion criteria*

The literature search for this systematic review specifically targeted peer-reviewed articles and other relevant literature that extensively discussed and provided insights into Primary Health Care (PHC) services and their delivery mechanisms. The aim was to encompass scholarly works that comprehensively addressed PHC systems, policies, interventions, and their impact on population health outcomes. Inclusion was based on the relevance of the content to the study’s objective of analyzing and synthesizing information related to PHC’s role in fostering a healthy population. The selected studies were expected to contribute substantial data and insights pertinent to the systematic review conducted in this review.

*Exclusion criteria*

Conversely, articles falling outside the scope of addressing the designated topic or failing to furnish relevant data essential for the systematic review were excluded. This entailed excluding studies that did not directly delve into the realm of PHC services or failed to provide substantial and pertinent information aligned with the study’s objectives. Additionally, articles not published in the English language were excluded from this review to ensure the consistency and comprehensiveness of the analysis conducted within the confines of accessible and comprehensible literature.

The rationale behind these criteria was to focus on scholarly works that significantly contributed to the understanding of PHC services and their impact on population health outcomes while ensuring a level of uniformity and accessibility by restricting the language of publication to English.

*Data extraction*

The data extraction process involved screening the titles and abstracts of articles retrieved from the literature search. Full-text articles that met the inclusion criteria were considered for data extraction. We discussed the PHC services provided, identified gaps and improvement strategies from the retrieved articles.

### Data items

*Outcomes sought*

• For this systematic review, data were sought for several specific outcomes related to Primary Health Care (PHC) services, including but not limited to:
• Health outcomes: Measures encompassing various health indicators, such as mortality rates, disease prevalence, quality of life assessments, and healthcare utilization metrics.
• Service delivery gaps: Identification of gaps in access, affordability, and quality of PHC services as reported in the literature.
• Implementation strategies: Insights into strategies adopted for PHC implementation, including community involvement, infrastructure improvements, and healthcare workforce development.
• It is important to note that while efforts were made to collect all results compatible with each outcome domain in each study, certain limitations existed due to varied reporting standards across studies. Methods used to decide which results to collect involved prioritizing outcomes based on relevance to the study’s objectives and the availability of clear and comprehensive data.

*Other variables sought*

• Participant and intervention characteristics: Data sought encompassed demographic information (age, gender), geographic location, intervention types, and characteristics of PHC systems studied.
• Funding sources: Information regarding funding sources for the studies under review was sought to ascertain any potential biases or conflicts of interest.
• Assumptions about missing or unclear information were made cautiously. In cases of missing or ambiguous data, assumptions were made based on available contextual information and considering the impact of such missing data on the overall analysis and interpretation of results.

### Study risk of bias assessment

In conducting the systematic review, a comprehensive evaluation of potential biases within the included studies was undertaken using established criteria relevant to the respective study designs. The risk of bias assessment aimed to ensure the reliability and validity of the synthesized evidence.

1. Publication bias mitigation: To minimize publication bias, efforts were made to include both published and unpublished studies. Comprehensive search strategies across multiple databases and the inclusion of grey literature aimed to capture a wide array of research, reducing the likelihood of bias due to selective publication.
2. Selection bias considerations: The inclusion and exclusion criteria were explicitly defined to limit selection bias. The rationale behind study selection was based on pre-defined criteria to ensure a representative sample of studies addressing primary health care, thus reducing the risk of bias related to specific populations or study types.
3. Quality and consistency assessment: Studies included in the review underwent a rigorous quality assessment process. The Cochrane Risk of Bias Tool (or other appropriate tool based on study designs) was applied to assess individual study quality. This involved examining domains such as randomization, blinding, allocation concealment, and other relevant aspects to gauge the risk of bias in each study.
4. Reporting biases: Efforts were made to identify and address reporting biases by scrutinizing available data, looking for discrepancies between reported and unreported findings within the included studies.

Through this systematic and methodical approach, the review aimed to provide an accurate and comprehensive synthesis of the available literature, accounting for potential biases within individual studies to strengthen the validity of the collective conclusions. To process the results, 2 reviewers worked independently to avoid the risk of bias.

### Data synthesis and analysis

Data synthesis and analysis includes identifying gaps in PHC service delivery. Data were synthesized through a narrative synthesis of findings from articles included in the systematic review. The findings were summarized according to the provided medical services, identified gaps and improvement strategies.

By summarizing and qualitatively comparing the studies we conducted, we used a combination of documentary analysis and narrative synthesis as an alternative to meta -analysis, which has practical value in the development of PHC.

Our analysis involved a comprehensive approach that went beyond simply synthesizing the data. We carefully considered the views, challenges, and recommendations outlined by the World Health Organization (WHO) and other health experts. This approach ensured that our narrative synthesis was not only informative but also nuanced and grounded in the latest thinking on primary healthcare. Specifically, we incorporated insights from WHO publications and the expertise of health specialists to strengthen the accuracy and credibility of our findings. By combining data analysis with established knowledge, we were able to present a more robust and impactful evaluation.

For a complete picture, we reviewed a total of 10 articles from the World Health Organization.

## Results

The systematic review of the last ten years of written research on primary health care (PHC) services revealed that while the PHC system is crucial in ensuring a healthy population, there are significant gaps in its delivery across countries. ^22^ These gaps significantly impact the health outcomes of individuals and communities, and the PHC system needs to be strengthened to address them effectively.

The review identified a range of PHC services provided across different countries, including preventive services, such as vaccinations, health education, and screening and curative services, such as treatment for acute and chronic conditions. Study found that consultation times in primary care are often too short, with many patients spending less than 5 minutes with their physicians. This is a concerning trend that can negatively impact patient care and outcomes. Short consultation times:18 countries results - Approximately 50% of population spends less than 5 minutes with their primary care physicians which is Negative impact on patient care: Inadequate consultation time can lead to poor health outcomes, including missed diagnoses, inadequate treatment, and reduced patient satisfaction. ^23^ Despite the variation in the services provided, several gaps were identified in delivering PHC services across countries. These gaps included inadequate staffing, limited access to essential medicines, insufficient funding, inadequate infrastructure, and inadequate training for health workers. ^17^^,^^24^

One of the significant gaps identified in the delivery of PHC services was inadequate staffing. ^25^ Many countries need more health workers, particularly in rural and remote areas with inadequate health facilities. The shortage of health workers affects the quality and accessibility of PHC services and leads to long waiting times, reduced patient satisfaction, and poor health outcomes. In addition, the shortage of health workers exacerbates the burden of communicable and non-communicable diseases, contributing to a high mortality rate. ^23^

Another significant gap identified in the delivery of PHC services was limited access to essential medicines. ^26^ Many PHC facilities in low- and middle-income countries face shortages of essential medicines, which affects the quality of care and health outcomes. The lack of essential medicines often leads to inappropriate treatment, prolonged illness, and complications, which significantly impact the quality of life of patients and their families.

Insufficient funding was another gap identified in the delivery of PHC services. ^2^ Many countries need more funding for the PHC system, which affects the quality and availability of services. The lack of funding leads to adequate infrastructure, adequate staffing, and limited access to essential medicines and medical equipment. As a result, patients face long waiting times, reduced patient satisfaction, and poor health outcomes.

Inadequate infrastructure was also identified as a gap in the delivery of PHC services. ^16^ In primary health care, the impact of access to medicines on quality of care is also important. The current study analyzes data from 45 WHO/HAI surveys conducted in 36 countries to assess the availability, affordability and cost of medicines and found evidence that this has a negative impact on poor health outcomes such as: non-adherence to treatment, Financial hardship, increased mortality. To address these challenges, countries should implement a number of policies and interventions, including implementation of price controls, promotion of generic drugs, strengthening of public procurement systems, strengthening of international cooperation, expansion of health insurance coverage, investment in domestic production of drugs. ^27^ Many PHC facilities need more basic amenities, such as clean water, electricity, and adequate sanitation facilities, affecting care quality and safety. The lack of infrastructure often leads to poor hygiene, increased risk of infection, and reduced patient comfort, which affects patient satisfaction and health outcomes. ^28^^,^^29^

A systematic review highlights the critical role of appropriate counseling and health education in providing evidence-based care. Despite the importance of these elements, many primary healthcare providers in low-income and middle-income countries (LMICs) are unable to offer adequate counseling on topics like pregnancy complications, HIV prevention, contraceptive side effects, and chronic disease management. To address these deficiencies and improve overall healthcare outcomes, there is a growing interest in strengthening primary care. ^29^ Primary care has traditionally been a cornerstone of healthcare in LMICs, but evolving disease patterns, urbanization, and increasing demand for specialized services are challenging the current model. Research indicates that many primary care providers do not follow management guidelines and may not be practicing prevention effectively. This underscores the need to balance access and quality in healthcare. While access is important, ensuring a minimum level of quality for all is essential for achieving equitable health outcomes and avoiding wasted resources. To improve healthcare quality, it is crucial to measure and report on health system performance. This includes tracking health outcomes, user experience, and system competence. However, existing data often lacks the necessary depth and breadth to inform decision-making and guide improvements. Efforts to strengthen primary care and improve healthcare quality must be comprehensive and address both the individual provider level and the broader healthcare system. By focusing on evidence-based practices, enhancing counseling skills, and addressing systemic issues, LMICs can make significant strides in improving the quality of care provided to their populations. ^29^

Finally, inadequate training for health workers was identified as a gap in the delivery of PHC services. ^30^ Many health workers need more skills and knowledge to provide quality care, particularly in managing non-communicable diseases. The lack of training leads to poor diagnosis, inappropriate treatment, and reduced patient satisfaction, which affects health outcomes.

Despite ongoing efforts to enhance chronic disease management, the global mortality rate from these conditions remains high. This research has interesting findings—proposing a conceptual framework for a chronic care model (CCM)—to improve care for patients with chronic diseases. The core components of CCM are self-management support, delivery system design, decision support, clinical information systems: electronic health records and Using other information systems to support chronic care. ^28^

With the help of primary care services, patients are under constant monitoring and the doctor observes the patient throughout his life. As a result, the doctor has complete information about the patient’s disease, how it developed and how it is developing. This allows the doctor to better manage the disease. Another distinctive feature of primary care is the comprehensiveness of care because the family doctor cares not only for the patient’s physical, but also for his spiritual and social well-being. Health care is a complex system that also has a kind of coordination function.

The family doctor coordinates the full service of the patient, and he is not only a doctor but also a supporter, guide and coordinator who can protect patients and help them make the right choice during medical care. Through the doctor’s coordination, the patient receives the appropriate service at the appropriate time and place. ^1^

### Search and selection process

The systematic review commenced with an initial search across electronic databases (PubMed et al. Library and Web of Science) to identify relevant articles. A total of [57 articles] records were identified through the search process. Following removing duplicates, [47 articles] unique records underwent title and abstract screening. Subsequently, [38 articles] full-text articles were assessed for eligibility based on predefined inclusion criteria.

### Studies included and excluded

Throughout the review process, studies that appeared to meet the inclusion criteria were assessed; however, [14 articles] studies were excluded due to -irrelevant study design, inadequate data.

### Characteristics of included studies

The systematic review included [38 articles] studies that met the predefined inclusion criteria. Each included study’s characteristics, including participant demographics, study design, interventions, and outcomes measured.

*Presentation of outcomes*

The review synthesized data from included studies of gaps in PHC services. Critical gaps identified encompassed inadequate staffing, limited access to essential medicines, insufficient funding, inadequate infrastructure, and insufficient training for health workers.

## Discussion

The gaps identified in the delivery of PHC services significantly impact the health outcomes of individuals and communities, especially in low- and middle-income countries. This study, however, has limitations. Firstly, the literature search was confined to articles published in English, potentially excluding relevant information published in other languages. Secondly, the quality of the included articles might have constrained the systematic review findings, urging caution in their interpretation.

Despite these limitations, the study conducted a systematic review over the past decade, examining written research on healthcare services utilizing peer-reviewed articles and pertinent literature on PHC services. The study’s primary objective was to identify PHC service gaps and propose strategies to improve health outcomes.

Comprehensive and coordinated strategies targeting the root causes are imperative to address these identified gaps effectively. Policy interventions addressing staffing shortages, increased funding for the PHC system, infrastructure enhancement, and adequate training and support for healthcare workers are critical.

A pivotal strategy involves bolstering health systems, necessitating the formulation of policies and programs enhancing the quality, accessibility, and affordability of health services. Achieving this may entail appropriate financing mechanisms, robust health governance systems, bolstered health workforce, and refined health information systems.

Furthermore, integrating PHC services into the broader health system is another crucial strategy. This integration can bridge gaps and synergize efforts, ensuring more comprehensive and efficient healthcare delivery.

The US spends the most on healthcare per capita but has poor health outcomes. This could be due to factors like the fee-for-service model that incentivizes more procedures instead of preventive care. Team-based care and technological integration are crucial for transforming primary healthcare. While many practices already utilize teams, a higher level of collaboration is required. ^21^ This involves defining new roles, partnering with social services and other specialties, and adopting a patient-centered approach. Additionally, traditional hierarchical structures within healthcare delivery must adapt to support these team-based models. Integrating technology is essential. Patient-facing technologies, such as patient portals, data collection tools, and personalized coaching, can enhance patient satisfaction, improve efficiency, and elevate care quality. For instance, patient portals enable asynchronous communication with care teams, leading to greater convenience and better outcomes. ^21^ The study was conducted in 31 countries (including 27 EU members States, Iceland, Norway, Switzerland and Turkey) - A study was conducted to assess The strength of primary care in Europe and although many countries prioritize the development of primary care, ^31^ there are challenges that need to be addressed: Strengthening primary care governance: establishing a clear government vision and policy for primary care, ensuring effective governance and accountability Improving economic conditions. Addressing income inequality for primary care providers, particularly in Eastern Europe Investing in workforce development. Addressing workforce shortages by investing in education and training of primary care professionals. Increasing access. Implementing policies to improve geographic access to primary care Promoting continuity of care Implement patient registration systems and encourage long-term relationships between patients and primary care providers. Leverage technology - Equip primary care practices with adequate technology to support efficient operations and improve communication. Enhance patient-provider relationships - Focus on improving communication skills and building trust between primary care providers and patients on formation.

## Conclusion

Primary health care (PHC) is crucial in ensuring a healthy population and addressing global health challenges. However, there are significant gaps in the delivery of PHC services across countries, which significantly impact the health outcomes of individuals and communities.

Our study revealed several gaps in the delivery of PHC services, including inadequate staffing, limited access to essential medicines, insufficient funding, inadequate infrastructure, and inadequate training for health workers. ^27^^,^^32^ These gaps affect the quality and accessibility of PHC services, and lead to long waiting times, reduced patient satisfaction, and poor health outcomes.

To address these gaps effectively, comprehensive, and coordinated strategies are needed that address the underlying causes of these gaps. These strategies should include policies to address staffing shortages, increase funding for the PHC system, improve infrastructure, and provide training and support for health workers. ^1^^,^^32^

Strengthening health systems is a key strategy for addressing the gaps in PHC services. This involves the development of policies and programs that improve the quality, accessibility, and affordability of health services. This can be achieved through the development of appropriate financing mechanisms, the establishment of effective health governance systems, the expansion of the health workforce, and the improvement of health information systems. ^31^

Integrating PHC services into the broader health system is another strategy for addressing the gaps in PHC services. Integration involves coordinating services across distinct levels of care and involving different stakeholders, including governments, healthcare providers, and communities. This can help to improve the quality and accessibility of services, reduce duplication and fragmentation of services, and enhance the efficiency and effectiveness of the health system. ^33^^,^^34^

In conclusion, this study highlights the importance of PHC services in ensuring a healthy population and addressing global health challenges. It identifies significant gaps and challenges facing the PHC system and provides strategies for improving PHC services in practice. Studies have shown that among 90 countries with a gross national income of less than $10,000 per person, those that shifted to primary care and 30 such countries showed better health outcomes, reduced mortality, and concluded that increasing primary care physicians is associated with: 1.44 fewer deaths per 10,000 population , 2.5% reduction in infant mortality, 3.2% reduction in underweight, ^25^ countries that moved to universal primary health care achieved lower under-five mortality, more equitable distribution of health care, lower rates of breast cancer, reduced hospital use in these countries: 6% less inpatient admission, 5% fewer outpatient visits, 10% fewer emergency room visits, 7% less operations - Lower health care costs - these data are supported by strong studies that further support the evidence for the need to strengthen primary care. ^25^

These strategies are essential for achieving universal health coverage and addressing the Sustainable Development Goals related to health. Implementing these strategies requires the commitment and collaboration of all stakeholders, including governments, healthcare providers, and communities, to deliver high-quality, accessible, and affordable PHC services for all. ^35^^,^^36^

The current overview consolidates evidence supporting the development of primary healthcare (PHC) and the creation of a patient-oriented PHC system. To achieve this, it is essential to involve the public, gain political support, and focus on developing a model tailored to the needs of patients with multimorbidity. Prioritizing public education and enhancing self-esteem are crucial steps in this process.

Additionally, establishing home care services and providing organizational support can help reduce the burden of hospitalization. Developing information technology is also vital, as it will significantly improve the quality of care, facilitate tracking of health services, and positively impact health processes and outcomes.

This study, which is a scoping review, also highlights the need for further research into innovative health policies and programs to maintain a healthy population. ^37^

In 2022, the Chinese government prioritized increasing investment in primary healthcare (PHC) improvements, signaling to the international community that PHC is a crucial pathway to achieving a healthy population. This aligns with a significant study that applied an improved three-stage model (Data Envelopment Analysis, DEA) using the Global Benchmarking Technique (GBT) to evaluate China’s PHC system.

The large-scale study demonstrated that the availability of medical services is directly linked to improvements in PHC, underscoring the need for greater investment in its development. The findings revealed that PHC efficiency positively impacts population health. However, the study also showed a decline in three key indicators of PHC efficiency between 2010 and 2021: Total Efficiency (TE) decreased from 0.807 to 0.546, Pure Technical Efficiency (PTE) dropped from 0.870 to 0.594, and Scale Efficiency (SE) fell from 0.934 to 0.917.

These declines indicate a deterioration in performance, suggesting that increased financial investment and favorable policies alone are insufficient for the successful functioning of PHC. To address this, it is necessary to focus on:

• Effective allocation of resources
• Technological advancements
• Structural issues, including personnel and infrastructure ^2^

In the Current article While the study focused on data from 1980-1995, its findings remain highly relevant today. This research demonstrates the enduring importance of primary care, particularly family medicine, in improving population health outcomes, as evidenced by its significant association with lower mortality rates. These findings align with our systematic review, further reinforcing the value of primary care as a cornerstone of healthcare systems. ^33^

### Recommendations

1. Increase funding for primary health care: Adequate funding is essential for delivering quality primary health care services. Governments should increase their investment in the primary health care system, allocate sufficient resources, and ensure they are used efficiently and effectively. This would enable the system to address the gaps in the delivery of PHC services and ensure that all individuals have access to quality healthcare services.
2. Expand the health workforce: The shortage of trained health workers is a significant barrier to delivering quality primary health care services. Governments should develop policies and programs that increase the number of health workers in the system, improve their skills, and ensure they are deployed effectively. This would improve the availability and accessibility of primary health care services, particularly in underserved areas. Physician assistants can assume 25% of a general practitioner's workload, increasing interest in their role alongside nurses in primary care delivery. This ultimately contributes to the quality of primary care services.
3. Strengthen health information systems: Accurate and reliable health information is essential to deliver primary health care services effectively. Governments should invest in developing and strengthening health information systems that capture relevant health data and enable effective monitoring and evaluation of the PHC system. This would provide policymakers and healthcare providers with the information they need to make informed decisions and improve the quality of healthcare services in order to increase the convenience of patients in primary health care facilities.
4. Promote community participation: Community participation is essential for effectively delivering primary health care services. Governments should promote community participation in designing, delivering, and monitoring PHC services. This would enable the system to be more responsive to communities’ health needs and preferences and ensure that services are designed to meet their needs.
5. Foster intersectoral collaboration: Health is influenced by a range of factors, including social, economic, and environmental determinants. Governments should foster intersectoral collaboration across different sectors to address the underlying causes of health inequities and promote health equity. This would involve working with different sectors, such as education, housing, and transportation, to create supportive environments for health and improve the social determinants of health.
6. Embrace technological innovations: Technological innovations, such as telemedicine and electronic health records, can potentially transform the delivery of primary health care services. Governments should embrace these innovations and invest in their development and implementation. This would enable the system to be more efficient, effective, and responsive to the needs of patients.
7. Foster international collaboration: Primary health care is a global challenge that requires international collaboration and cooperation. Governments should collaborate, share best practices, and learn from each other’s experiences to improve the delivery of primary healthcare services. This would enable countries to work together to address global health challenges and achieve universal health coverage.
8. In order to promote the development of the Institute of Family Physicians, it is necessary to ensure the normal payment of primary health care personnel. It is advisable to introduce a combined method of primary care, which will increase their care.
9. Primary health care is the foundation of the health care system, the health quality of the population, access to services, effective spending of scarce funds allocated for health care significantly depend on the health care system. management and analysis of the epidemiological situation, and establishment of a healthy lifestyle. The main criterion of public health priority lies. primarily in the cost efficiency of preventive measures.
10. Implementation of PC Monitor (Primary care monitoring system) in countries to collect and compare data on primary care systems across Europe to the benefit of all countries. Regular implementation of PC Monitor (e.g. every 5 years) can track trends in primary care over time.
11. Redesigning microsystems: Small units of care delivery should be knowledge-based, patient-centered, and systems minded. Strengthening organizations: Healthcare organizations should be better designed to support microsystems and achieve the six aims (Patient-Centered Care, Safety, Effectiveness, Efficiency, Equity, Timeliness) for improvement. Reforming the external environment: The external environment of healthcare, including financing, regulation, accreditation, litigation, professional education, and social policy, should be more conducive to quality improvement.
12. Promote team-based care: This approach can improve quality of care, increase patient satisfaction, and reduce costs. Value-Based Care, Encourage collaboration between primary care providers, specialists, and other healthcare professionals. Team-based care and technological integration are crucial for transforming primary healthcare. Community-Based Approach: Foster a community-based approach to primary healthcare, involving communities in decision-making and service delivery.
13. Patient Education: Provide patients with education and support to manage their health conditions effectively.

In conclusion, delivering quality primary health care services is essential for ensuring a healthy population and addressing global health challenges. Governments must prioritize developing and implementing policies and programs that address the gaps in delivering PHC services and ensure that all individuals have access to quality healthcare services. This requires a commitment to collaboration, innovation, and investment in the PHC system to achieve universal health coverage and address the Sustainable Development Goals related to health.

Our systematic review examined the role of primary health care (PHC) in maintaining a healthy population and the challenges faced by different countries in implementing PHC. We found a growing interest in systemic PHC reforms, aligned with the World Health Organization's (WHO) goal of universal health coverage (UHC) by 2030.The WHO's Declaration on Universal Health Coverage emphasizes a patient-centered approach that covers the full range of essential health services. ^38^ By investing in primary care, countries can improve health outcomes, save lives, and increase life expectancy. Our review confirms that PHC is crucial for a healthy population. While many articles highlight challenges and negative impacts of weak PHC, they also offer recommendations for improvement. (See Table 1 for details.) To develop effective primary care systems, countries need to implement fundamental reforms. Health experts worldwide recognize the importance of primary health care and the need for change. Studies have shown that current approaches are insufficient to provide patient-centered care.

Urgent action and investment are necessary to achieve a well-structured PHC. Our article calls for further research on concrete steps to achieve this goal. Rudolf Virchow's influential statement, "Medicine is a social science and politics is nothing but medicine on a large scale," continues to be a cornerstone of public health. This perspective highlights the critical role of social factors in determining health outcomes and underscores the need for a comprehensive approach to healthcare management.

While significant progress has been made in understanding the social determinants of health and the importance of primary care, developing a universally applicable management model for healthcare remains a complex challenge. This is due to the diverse range of factors influencing healthcare systems, including cultural, economic, and political contexts. ^34^

In conclusion, Strengthening Primary Healthcare for a Healthier World.

The 2019 United Nations Declaration on Universal Health Coverage (UHC) reinforces the pivotal role of primary healthcare in achieving sustainable development and improving population health. As outlined in the declaration, primary healthcare serves as the cornerstone of a resilient and people-centered health system, offering accessible, affordable, and quality care to all. This further strengthens our review and proves that strong, well-structured primary health care is indeed the guarantor of a healthy population. ^35^

## Data Availability

All data underlying the results are available as part of the article and no additional source data are required. Figshare: PRISMA-S checklist for ‘ *Primary Health Care as The Main Guarantor of a Healthy Population in The Country and A Global Challenge in The World” a systematic review*’, https://doi.org/10.6084/m9.figshare.24846126.v1. ^39^ Data are available under the terms of the Creative Commons Zero “No rights reserved” data waiver (CC0 1.0 Public domain dedication).
