# Supplementary material for: Primary health care as the main guarantor of a healthy population in the country and a global challenge in the world: a systematic review
Source: F1000Res. 2024 Oct 10;13:38. Originally published 2024 Jan 8. [Version 2] doi: 10.12688/f1000research.141252.2 (PMC12231440; doi:10.12688/f1000research.141252.2)
Supplement: Peer review history

# Reviewer report: Reviewer response for version 2

**DOI:** 10.5256/f1000research.170641.r376802

**Published:** 2025-05-09

**Authors:** Augustino Mwogosi

**Affiliations:** 1 The University of Dodoma, Dodoma, Dodoma Region, Tanzania

The manuscript  presents a timely synthesis of existing global research on primary health care systems. The authors aim to explore the relationship between PHC structures and population health outcomes, identify service delivery gaps, and offer recommendations to enhance PHC implementation, particularly in the context of Georgia, where PHC reform remains a pressing issue. Using a combination of documentary analysis and narrative synthesis, the study reviews 38 articles (including 6 older papers for historical context) to highlight recurring challenges in PHC delivery, such as affordability, accessibility, and system fragmentation. The study follows PRISMA guidelines and presents a summarized data extraction table and a PRISMA flow diagram. However, there are some areas that need improvements

**1. Search strategy details** are vague. Exact Boolean search strings for each database are not provided.

 2. The **study selection process** is not described in enough detail (example: whether a third reviewer resolved conflicts, if software was used, etc.).

 3. There is **no description of critical appraisal tools** used to assess study quality.

 4. No **protocol registration** (e.g., in PROSPERO) is reported, which is essential for transparency and bias prevention.

 5. The flow diagram does not provide sufficient detail (numbers of records retrieved, removed, etc., also reasons for exclusions at the full-text level, handling of duplicates).

**Recommendations to Improve Scientific Rigor:**

 1. Include full **search strings** in the appendix or methods section for reproducibility.

 2. Clarify the **screening process**, including how discrepancies between reviewers were resolved.

 3. State whether **any formal tool** (e.g., CASP, MMAT) was used to appraise study quality; if not, explain the rationale.

 4. Clearly declare whether the review protocol was registered, and if not, provide a justification and suggest retrospective registration if possible.

 5. Stating the **number of duplicates removed**

 Although statistical analysis is not applicable, the authors should **enhance the transparency** of the **data synthesis process**, for example, by explaining how themes or categories were derived and providing more structure to the narrative (for example, grouping findings into themes such as financing, service delivery, and governance).

 The manuscript covers a globally important topic and presents valuable insights into PHC reform, especially in low-resource settings like Georgia. It contributes meaningfully to international health systems research. However, to make the article scientifically sound and replicable, **minor but essential revisions** are required particularly concerning **methodological transparency, PRISMA adherence, and protocol registration**.

Are the rationale for, and objectives of, the Systematic Review clearly stated?

Yes

Is the statistical analysis and its interpretation appropriate?

Not applicable

If this is a Living Systematic Review, is the ‘living’ method appropriate and is the search schedule clearly defined and justified? (‘Living Systematic Review’ or a variation of this term should be included in the title.)

Not applicable

Are sufficient details of the methods and analysis provided to allow replication by others?

Partly

Are the conclusions drawn adequately supported by the results presented in the review?

Yes

Reviewer Expertise:

My research centers on health informatics and digital health systems, with a particular emphasis on the implementation and evaluation of electronic health records (EHR) in low- and middle-income countries. I am especially interested in decision support systems and the application of emerging technologies, including artificial intelligence, in enhancing health service delivery. Within the field of health informatics, my work also examines strategies for strengthening health systems through data-driven primary healthcare reforms, informatics-based monitoring and evaluation of health programs, and the responsible use of AI in personal data protection and digital transformation of healthcare services.

I confirm that I have read this submission and believe that I have an appropriate level of expertise to confirm that it is of an acceptable scientific standard, however I have significant reservations, as outlined above.

# Reviewer report: Reviewer response for version 2

**DOI:** 10.5256/f1000research.170641.r366910

**Published:** 2025-02-17

**Authors:** Shalva Zarnadze

**Affiliations:** 1 Department of Nutrition, Aging Medicine, Environmental and Occupational Health, Tbilisi State Medical University, Tbilisi, Georgia

**1. Summary of the Article**

 This systematic overview by Abashidze-Gabaidze et al. explores the place of primary health care (PHC) as an anchor for an unhealthy-free society, both locally and globally. The writers address the opportunities and threats confronting strengthened PHC system building, focusing on implications for policymakers and practical advice. The study reviews the synthesis of evidence from several sources that indicate PHC's relevance to achieving equitable health gains and sustainable development. The study is relevant specifically to Georgia and the region of the Caucasus, whereby PHC system building has clear opportunities and threats due to limited resources, as well as historical context.

**2. Strengths of the Article**

**Strengths Relevance and Timeliness**: The paper touches on an incredibly relevant, time-specific topic whereby primary health care have risen as an invaluable building brick of health systems globally. The focus on Georgia, as well as the region of the Caucasus, adds immense worth, considering the clear opportunities confronting this region.

**Comprehensive Methodology:** Mixed-methods approach, narrative synthesis, complemented by documents' analysis, best suited to analyze complexities of PHC. Including recent (2002–2023) studies add credibility. A vast overview over many years confirms that primary health care does not disappear. However, it has not emerged - the gold standard, strengthening it - so this extensive-scale overview makes scope, analysis, and comparison possible for recommendations. In this, the authors have - a Prisma diagram of study selection, PRISMA Checklist, added, additionally, Table N1, showing the level, expertise, responsibility, diligence while preparing the paper, focusing, as per importance, need, importance, by evaluating, strengthening, prioritizing, first, primary health care by nations, including Georgia, also by greater well-being, by reviewing, analyzing, studying 38 papers over primary health care, prepared over many years, over many nations, additionally, confirming importance, relevance, of the paper, that strengthening, enhancing, primary health care must remain an agenda both over Georgia, also over general system, over healthcare, over the whole world.

**Practical Recommendations:** The paper makes implementable, practical recommendations, recommendations especially by policymaking authorities, especially over low- and middle-income nations (LMICs) like Georgia. These areas, especially staffing, funding, and infrastructure, are beneficial.

**Global, Local Context:** The overview balances well both global priorities (for example, the importance accorded by WHO over universal health coverage) and local issues, so it serves well both international and local stakeholders.

**3. Weaknesses, Areas Improvable**

**Important Points:**

**Limited Access to Information:** Authors are aware of the problem posed by the inability to attain access to detailed, accurate information, especially in Georgia. Although this must have been due to the region's limitations, it would have benefited if there had been an increased description of how these limitations affected findings and recommendations.

**Limitations of Narrative Synthesis: **Although narrative synthesis fits this type of overview, lack of meta-analysis may not always make it possible to offer sound statistical findings. However, this is only minor due to the qualitative study.

**Minor Points:**

**Specific Detailing Recommendations: **Recommendations concerning implementation strategies, especially by Georgia, are vague. An example includes how proposed policies must be localized by considering the limitations of the local environment.

**4. Relevance and Contribution**

**Contribution to Literature**

 The paper contributes well to the burgeoning corpus of work relating to primary health care, especially in Georgia and the region of the Caucasus. Findings are relevant to policymaking and researchers and practitioners are intent on developing strengthened health systems. Practical recommendations add substance to the paper, especially if they have implications for areas similarly challenged.

**5. Final Recommendation**

 This is an exhaustively conducted systematized review of an absolutely fundamental topic. Despite limitations due to data unavailability, coupled with the adversities experienced by Georgia, the authors have conducted an exhaustive, perceptive analysis. Minor flaws are not enough to blemish the quality and worthiness of the paper. Hence, this paper is very strongly recommended for indexing. It shall be an excellent addition to the field and an invaluable tool, and it shall remain so for scholars, policymakers, and practitioners involved in strengthening PHC across the region. Apart from minor criticisms, this work shall be handy and one of the best across the region, especially in Georgia. Papers like this must get indexed so that they act as an encouragement factor to make other scholars think beyond the scope of this paper while widening the scope of their work.

**Further Comments**

**Acknowledging Limitations**: Appreciation must be accorded here; it must be noted that the adversities experienced by the authors while carrying out this review, especially owing to data unavailability coupled with insufficiency across Georgia, must be appreciated. These limitations are endemic across LMICs, and does this deem down the relevance across findings? Instead, these underscore the need for increased international support, especially coordination, to strengthen the PHC system across these nations.

**Value across Georgia**: The paper is especially valuable across Georgia, as it has an agenda of bridging PHC service deficits. The recommendations are practical, localised, and, thus, very actionable across policymaking circles and the healthcare service delivery system.

**Conclusion**

 This systematic analysis adds significantly to the literature review on strengthening PHC, particularly for Georgia and the Caucasus region. Given writers' challenges, this study has practical implications and sound contributions toward strengthening the PHC system. The paper has minor flaws that outweigh these, which warrants my highest recommendation.

Are the rationale for, and objectives of, the Systematic Review clearly stated?

Yes

Is the statistical analysis and its interpretation appropriate?

Yes

If this is a Living Systematic Review, is the ‘living’ method appropriate and is the search schedule clearly defined and justified? (‘Living Systematic Review’ or a variation of this term should be included in the title.)

Yes

Are sufficient details of the methods and analysis provided to allow replication by others?

Yes

Are the conclusions drawn adequately supported by the results presented in the review?

Yes

Reviewer Expertise:

Public health

I confirm that I have read this submission and believe that I have an appropriate level of expertise to confirm that it is of an acceptable scientific standard.

# Reviewer report: Reviewer response for version 2

**DOI:** 10.5256/f1000research.170641.r330790

**Published:** 2024-11-15

**Authors:** Richard Baker

**Affiliations:** 1 Department of Health Sciences, University of Leicester, University of Leicester, England, UK

The revisions to the paper have helped clarify what the authors did or did not do and brought to light the need for further revisions. The authors have undertaken a lot of work, but the additional revisions required are entirely manageable. 

 My principle points are:

 1. This is not a systematic review, but could reasonably be described as a selective review. The literature search, the inclusion and exclusion of articles, and the narrative synthesis all indicate a selective rather than systematic approach. If, as stated in the introduction, the overall aim is to provide guidance to the development of primary health care in Georgia, a selective review is reasonable. It enables the authors to select evidence and messages tailored to the prevailing situation in Georgia.

 2. The article needs a careful edit. There is quite a bit of duplication and various grammatical errors that could be usefully corrected. Could the Journal editors help the authors with this?

 Further detailed points are included below.

 Introduction

 A reference is needed for the statement that 80% of global deaths are attributable to chronic conditions.

 A reference is also needed to support the statement that 90% of UHC interventions can be implemented through primary health care (PHC). Both these statements may be over-stating the case.

 The outline of the problems of PHC in Georgia is a welcome addition and leads in to the aim of the paper – to develop recommendations to improve PHC in Georgia. Some words are missing from the subsequent paragraph, highlighted with square brackets. 

 The introduction offers a broad overview of PHC. Mention of WHO’s definition and view of PHC is relevant, but there is a lot of duplication in the following paragraphs that could usefully be omitted.

 Methods

 The search strategy is described and appears extensive. The inclusion of a copy of the strategy for each bibliographic database is unnecessary, but it would be wise to include in a supplementary at least on search strategy as an example, ideally PubMed. The PRISMA chart indicates that 57 articles were identified from the database searching. This is a very low number if it refers to the articles listed in the databases – the number of hits relating to primary health care would be expected to be many thousands. Some explanation of the small number of articles included is needed. The inclusion and exclusion criteria were quite broad and it’s possible that they allowed room for subjective judgments about an article’s eligibility for inclusion. The selection of articles, according to the inclusion/exclusion criteria, were influenced by whether they were regarded as ‘pertinent’, an approach that should not be regarded as systematic.

 The Table of included studies is helpful. It describes each of the studies clearly, although only 27 articles were in the Table but 38 were included in the review. They are a heterogenous group of articles, both in terms of design and topic. Some of the articles are discussion or opinion pieces; for example, reference 34 is a commentary and reference 9 is a book that describes the US health care system. This means that the review is not systematic. I’m not clear how risk of bias tools would have been applied to these discussion pieces. A narrative synthesis was undertaken, a suitable approach given the nature of the papers that were included. 

 The Results section draws out many of the weaknesses in the delivery of PHC in various countries. Some information on the included articles is presented at the end of the results section but a summary of the numbers of hits and articles included should be at the start of the results section.

 A Conclusions section provides further evidence, and a set of 13 recommendations is then presented. The conclusions section could be re-drafted to concentrate on what needs to be done next to strengthen PHC rather than summarize findings from a few of the included papers. The recommendations may well be directed at the health system in Georgia although this is not stated explicitly. If this is intended, it should be made clear. Ending the paper on messages for the Georgia health system would tie in well with the remarks in the introduction.

Are the rationale for, and objectives of, the Systematic Review clearly stated?

Yes

Is the statistical analysis and its interpretation appropriate?

Not applicable

If this is a Living Systematic Review, is the ‘living’ method appropriate and is the search schedule clearly defined and justified? (‘Living Systematic Review’ or a variation of this term should be included in the title.)

Not applicable

Are sufficient details of the methods and analysis provided to allow replication by others?

Partly

Are the conclusions drawn adequately supported by the results presented in the review?

Partly

Reviewer Expertise:

primary care and population mortality

I confirm that I have read this submission and believe that I have an appropriate level of expertise to confirm that it is of an acceptable scientific standard, however I have significant reservations, as outlined above.

# Reviewer report: Reviewer response for version 2

**DOI:** 10.5256/f1000research.170641.r330791

**Published:** 2024-10-12

**Authors:** Nia Roberts

**Affiliations:** 1 University of Oxford, Oxford, England, UK

As an information specialist I can only comment on the search methods. In comparing what's reported in the methods section of the paper with the reporting requirements of PRISMA 2020 in relation to:

 6. Information sources: 

 Specify all databases, registers, websites, organisations, reference lists and other sources searched or consulted to identify studies. Specify the date when each source was last searched or consulted.

 7. Search strategy:

 Present the full search strategies for all databases, registers and websites, including any filters and limits used.

 This paper does not fulfill the reporting  requirements to be regarded as a systematic review.

 There's no indication how the listed keywords were combined, therefore it isn't possible to assess whether potentially relevant papers were missed. The small number of papers retrieved across databases listed does not lead me to believe that a comprehensive search was conducted.

 The authors do not report when the search was conducted, but state that the search was limited to last 10 years. They have however, cherry picked articles published 2002-2008, and then included studies spanning 2010-2023. This inconsistency is highly irregular and would not be considered appropriate for a systematic review.

 I'm not a topic expert, so this paper may well be considered to be a well argued narrative literature review. From a methodological point of view, the search methods are not sufficiently reported for it to be considered a systematic review.

Are the rationale for, and objectives of, the Systematic Review clearly stated?

Partly

Is the statistical analysis and its interpretation appropriate?

I cannot comment. A qualified statistician is required.

If this is a Living Systematic Review, is the ‘living’ method appropriate and is the search schedule clearly defined and justified? (‘Living Systematic Review’ or a variation of this term should be included in the title.)

No

Are sufficient details of the methods and analysis provided to allow replication by others?

No

Are the conclusions drawn adequately supported by the results presented in the review?

No

Reviewer Expertise:

Information specialist, who is only able to comment on the appropriateness of the search methods.

I confirm that I have read this submission and believe that I have an appropriate level of expertise to state that I do not consider it to be of an acceptable scientific standard, for reasons outlined above.

# Response

**Published:** 2024-10-14

**Authors:** Gulnara Abashidze-Gabaidze

**Affiliations:** Public Health, Ivane Javakhishvili Tbilisi State University, Tbilisi, Tbilisi, Georgia

Ms. Nia Roberts, we are deeply grateful for your time and valuable advice. Your suggestions have been instrumental in strengthening our article. We have carefully considered your feedback and implemented your recommendations to the best of our ability.

 As you requested, we have included detailed information about the study characteristics in Table N1. Additionally, Figure N1 (PRISMA diagram) and the PRISMA checklist are provided to ensure the transparency of our narrative synthesis.

 We are sincerely thankful for your expert guidance, which will help us to improve our writing in the future.

# Reviewer report: Reviewer response for version 1

**DOI:** 10.5256/f1000research.154678.r272310

**Published:** 2024-05-17

**Authors:** Richard Baker

**Affiliations:** 1 Department of Health Sciences, University of Leicester, University of Leicester, England, UK

Overview

 Congratulations to the authors for making the case that primary health care is key to the effectiveness of health systems. Round the world, primary health care needs strengthening both to improve population health and to increase the efficiency of health services. 

 Although others have reviewed the effects of primary health care, this new study investigates gaps in care, in the lack of staff, shortages of funding, poor access to medicines, inadequate facilities and infrastructure, and inadequate training. The identification of these problems, shared by many countries, is important, and this constitutes a powerful message for policymakers – if they aim to improve the health of the people they are responsible for, they must strengthen primary health care.

 Specific comments

 The paper might be adjusted in a few ways to make it more accessible. 

 1. The Introduction covers a lot of relevant issues. Would it be worth saying something about primary health care in Georgia, if one aim of the paper is to inform the health system in George? Also, it is not clear why public health is given a paragraph on page 3. How does this fit in to the case for primary health care?

 2. It would be better to use the term systematic review rather than meta-analysis. Meta-analysis implies a quantitative or statistical approach to combining the findings; the paper is does not involve a statistical analysis and is really a systematic review. A narrative synthesis (page 6) is not a meta-analysis.

 3. The methods are generally well described and they indicate a careful approach was taken. It would be helpful to present more on the search strategy – would it be possible to include an example of the search strategy in a supplementary file?

 4. A PRISMA chart would be helpful, and could be included in the supplementary materials (https://www.bmj.com/content/372/bmj.n71). This would help readers understand at what stage and why papers were excluded from the review. A very wide range of outcomes was included, and this could have led to a vast quantity of papers – how were they reduced down to the number in the review?

 5. Care appears to have been taken in assessing the risk of bias in included studies. A standard format was used, for example. 

 6. The Results section needs to be re-ordered to first present basic information on the studies identified and the characteristics of those included. This information could be presented in a series of Tables that should show the country of origin, the outcomes of interest, and the findings of the assessment of risk of bias. Once this information has been reported, the findings can then be described. The findings themselves are interesting; the addition of tables describing the findings of each paper should help to explain the basis for the broad statements of the Results section.

 7. The discussion section mentions the strengths and limitations of the study, and sets out a series of conclusions. There is some repetition across the discussion/ conclusions/ recommendations, and some editing would help. The conclusions and recommendations themselves are all reasonable.

 8. The discussion might include a paragraph on how the review’s findings reflect those of other recent reviews of primary health care. This would help to show support the findings. 

 I hope the authors continue to conduct studies in this field. They have wishes for good luck in such an undertaking.

Are the rationale for, and objectives of, the Systematic Review clearly stated?

Yes

Is the statistical analysis and its interpretation appropriate?

Not applicable

If this is a Living Systematic Review, is the ‘living’ method appropriate and is the search schedule clearly defined and justified? (‘Living Systematic Review’ or a variation of this term should be included in the title.)

Not applicable

Are sufficient details of the methods and analysis provided to allow replication by others?

Partly

Are the conclusions drawn adequately supported by the results presented in the review?

Partly

Reviewer Expertise:

primary care and population mortality

I confirm that I have read this submission and believe that I have an appropriate level of expertise to confirm that it is of an acceptable scientific standard, however I have significant reservations, as outlined above.

# Response

**Published:** 2024-10-12

**Authors:** Gulnara Abashidze-Gabaidze

**Affiliations:** Public Health, Ivane Javakhishvili Tbilisi State University, Tbilisi, Tbilisi, Georgia

1. Thank you, **Mr. Baker, **for your thorough review and valuable feedback. Your insights have been instrumental in strengthening this article and ensuring its quality. I appreciate your time and expertise
2. Our primary objective was to collaborate with other countries, including Georgia, to gain insights from their experiences in PHC. In response to the World Health Organization's call, we aimed to unite countries in a global effort to address this disease. By forming an international partnership and fostering mutual cooperation, we sought to share knowledge and best practices.
3. I appreciate your thoughtful feedback. While I believe I could have further refined this text, I've found this experience invaluable for my future writing endeavors. Thank you for your guidance.
4. I've added a table describing the findings of each paper, which I think will help explain the rationale behind the broad statements in the results section.

**Are sufficient details of the methods and analysis provided to allow replication by others?**

         The data for each article reviewed during the analysis, including study characteristics, are presented in Table N1. Figure N1 (PRISMA diagram of study selection) and the PRISMA checklist are used to enhance the transparency of the narrative synthesis.

         Your feedback has been invaluable in strengthening this article. Thank you again.

# Reviewer report: Reviewer response for version 1

**DOI:** 10.5256/f1000research.154678.r263484

**Published:** 2024-05-15

**Authors:** Nia Roberts

**Affiliations:** 1 University of Oxford, Oxford, England, UK

I'm unable to assess the search methods employed in the conduct of this review as they are not reported in sufficient detail. The search strategies need to be available in full, In addition, the numbers need to be reported in a PRISMA flowchart indicating how many papers were retrieved in total, duplicates excluded, total screened on title/abstract, total screened full-text and reasons for exclusion.

 The authors consistently indicate that they have conducted a meta-analysis, but the results are presented as a narrative synthesis. I would have expected more papers to have been included to support the findings in the narrative synthesis, however, without view of the search process I can't decide whether relevant research might have been missed.

 The authors indicate that a risk of bias assessment was undertaken, however, I don't see a table reporting the assessment for each study.

Are the rationale for, and objectives of, the Systematic Review clearly stated?

Partly

Is the statistical analysis and its interpretation appropriate?

I cannot comment. A qualified statistician is required.

If this is a Living Systematic Review, is the ‘living’ method appropriate and is the search schedule clearly defined and justified? (‘Living Systematic Review’ or a variation of this term should be included in the title.)

No

Are sufficient details of the methods and analysis provided to allow replication by others?

No

Are the conclusions drawn adequately supported by the results presented in the review?

No

Reviewer Expertise:

Information specialist, who is only able to comment on the appropriateness of the search methods.

I confirm that I have read this submission and believe that I have an appropriate level of expertise to state that I do not consider it to be of an acceptable scientific standard, for reasons outlined above.
